# Optimal Dose and Safety of Intravenous Favipiravir in Hospitalized Patients With COVID‐19: A Dose‐Escalating, Randomized Controlled Phase Ib Study

**DOI:** 10.1002/cpt.70261

**Published:** 2026-03-18

**Authors:** Tim Rowland, Richard FitzGerald, Elizabeth Challenger, Laura Dickinson, Laura J. Else, Lauren Walker, Colin Hale, Victoria Shaw, Callum Kelly, Rebecca Lyon, Jennifer Gibney, Karim Dhamani, Margaret Irwin, Yvanne Enever, Michelle Tetlow, William Wood, Helen Reynolds, Justin Chiong, Orod Osanlou, Henry Pertinez, Katie Bullock, William Greenhalf, Andrew Owen, David G. Lalloo, Michael Jacobs, Julian A. Hiscox, Thomas Jaki, Pavel Mozgunov, Geoffrey Saunders, Gareth Griffiths, Saye H. Khoo, Thomas E. Fletcher, the AGILE CST‐6 Study Group, Shazaad Ahmad, Christopher J. Edwards, Lesley Dry, Georgie McKenzie, Aleksandra Ros, Michael Stackpoole, Laura Bradley, Karen Jennings‐Wilding, Nicholas Paton, Fred Hayden, Janet Darbyshire, Amy Lucas, Ulrika Lorch, Andrew Freedman, Richard Knight, Steven Julious

**Affiliations:** ^1^ Liverpool School of Tropical Medicine Liverpool UK; ^2^ Liverpool University Hospitals Foundation Trust Liverpool UK; ^3^ NIHR Liverpool Clinical Research Facility Liverpool UK; ^4^ Department of Pharmacology and Therapeutics, Institute of Integrative, Systems and Molecular Biology, Centre for Experimental Therapeutics (TherEx) University of Liverpool Liverpool UK; ^5^ PHARMExcel Welwyn Garden City UK; ^6^ Bangor University Bangor UK; ^7^ Centre of Excellence for Long‐acting Therapeutics University of Liverpool Liverpool UK; ^8^ Keble College Oxford UK; ^9^ Institute of Infection, Veterinary and Ecological Sciences, Faculty of Health and Life Science University of Liverpool Liverpool UK; ^10^ MRC Biostatistics Unit University of Cambridge Cambridge UK; ^11^ Faculty of Informatics and Data Science University of Regensburg Regensburg DE Germany; ^12^ Southampton Clinical Trials Unit University of Southampton & University Hospital Southampton NHS Foundation Trust Southampton UK

## Abstract

AGILE (NCT04746183) is a Phase Ib/IIa platform, evaluating candidates to treat COVID‐19. Candidate Specific Trial 6 evaluated the safety and optimal dose of a novel intravenous formulation of favipiravir in a dose‐escalating, open‐label, randomized, controlled, Bayesian adaptive Phase Ib trial. Hospitalized adults with PCR‐confirmed SARS‐CoV‐2 infection, within 14 days of symptomatic COVID‐19 were randomized 2:1 in groups of 6 (*n* = 4 favipiravir, *n* = 2 standard of care) to ascending doses of intravenous favipiravir twice daily (b.i.d.) for 7 days or standard of care. Clinical data, safety evaluations, virology and pharmacokinetic samples were collected. The primary outcome was safety. Secondary outcomes included clinical, pharmacokinetic and virological endpoints. Twenty‐four participants enrolled between September 10, 2022 and November 1, 2023 [10/24 female; median age 74 years (range 52–93)]. Favipiravir was well tolerated despite a high background rate of unrelated adverse events. No dose limiting toxicities were observed, with a model‐predicted dose limiting toxicity risk of 16.8% and probability of unacceptable toxicity of 2.7% at the highest dose level. No serious adverse events were deemed related to favipiravir but an expected association with asymptomatic, transient hyperuricemia was observed. Favipiravir exposures increased disproportionally to dose with significant accumulation in plasma, but with marked variability between participants within each cohort. This novel formulation of favipiravir was safe at sustained high doses that reached pre‐specified pharmacokinetic targets in a study group with frailty and complex health profiles. We consider doses up to 2,400 mg b.i.d. to be safe for further evaluation.

**Study Highlights**

**WHAT IS THE CURRENT KNOWLEDGE ON THE TOPIC?**

Pre‐clinical studies of favipiravir describe broad spectrum antiviral activity, positioning it as a therapeutic candidate for many RNA viruses. An oral formulation of favipiravir has been widely studied as a treatment for COVID‐19 in clinical trials. Pharmacokinetic modeling suggests the doses used in many of these trials may not have reached effective plasma concentrations.

**WHAT QUESTION DID THIS STUDY ADDRESS?**

Was a novel intravenous formulation of favipiravir safe and well tolerated in patients hospitalized with COVID‐19? Were pre‐specified pharmacokinetic parameters met at doses modeled to be effective for the treatment of COVID‐19?

**WHAT DOES THIS STUDY ADD TO OUR KNOWLEDGE?**

We demonstrate that high doses of intravenous favipiravir are safe and well tolerated in a frail and co‐morbid population who are likely to be eligible for antiviral treatment but are often excluded from early phase clinical trials. We characterize the pharmacokinetic profile of intravenous favipiravir at high doses and recommend an optimal dose to be used in future Phase II studies.

**HOW MIGHT THIS CHANGE CLINICAL PHARMACOLOGY OR TRANSLATIONAL SCIENCE?**

There is an ongoing unmet need for antivirals to effectively treat COVID‐19; however, the significance of these safety and pharmacokinetic data go beyond COVID‐19. High doses of favipiravir have a use case against RNA viruses with pandemic potential including viral hemorrhagic fevers and pandemic influenza. The intravenous formulation may be particularly relevant in severely unwell patients for whom oral dosing is not possible or in whom GI absorption is affected. The pharmacokinetic characterization from this study will be used to inform doses for use against a range of significant pathogens. Early phase clinical trials of IV favipiravir in Crimean‐Congo Haemorrhagic Fever are ongoing.

In the post‐pandemic phase of COVID‐19, novel directly‐acting antiviral therapeutics are still required. AGILE (NCT04746183, https://clinicaltrials.gov/study/NCT04746183) is a multi‐arm, multi‐dose, phase Ib/IIa platform using a Bayesian adaptive design, established to address this need.^1^ With a vaccinated population and the predominance of less pathogenic SARS‐CoV‐2 variants, patients hospitalized with symptomatic COVID‐19 are now overwhelmingly frail, co‐morbid, or immunocompromised. These groups are often excluded from early phase clinical trials.

Favipiravir (6‐fluoro‐3‐hydroxypyrazine‐2‐carboxamide; T‐705) is an antiviral, licensed in oral form for use against influenza in Japan, with broad spectrum capabilities in non‐clinical studies^2^ that have been investigated in clinical studies of patients with influenza,^3^, ^4^ Ebola virus disease^5^ and COVID‐19.^6^

Favipiravir is rapidly and completely absorbed following oral administration.^7^ Time to maximum plasma concentration is between 0.5 and 4.0 hours. The mean (SD) elimination halflife (*t*_1/2_) of favipiravir in plasma following single dose administration ranges from 1.3 (±0.1)–3.9 (±0.3) hours, depending on dose. Following multiple doses and at higher dose levels mean (SD) *t*_1/2_ of up to 8.7 (±5.1) has been demonstrated.^7^ Aldehyde oxidase (AO) is key in the metabolism of favipiravir; AO inhibition by favipiravir leads to accumulation of favipiravir in the plasma. Twice daily oral administration results in a greater than proportional rise in plasma concentration. Plasma protein binding averages 53–54%, 65% of which is due to albumin binding.^7^ The major metabolite, T‐705M1, is formed by AO in human liver cytosol and other tissues. The cytochrome mixed function oxidase systems do not play a significant role. Favipiravir and its metabolites T‐705M1 and T‐705M2 are excreted predominantly in urine, and to a lesser degree in feces. No dose alterations are needed in renal impairment. Favipiravir is intra‐cellularly phosphoribosylated to the active metabolite favipiravir ribofuranosyl‐5′‐triphosphate (T‐705‐RTP), which selectively inhibits RNA virus RNA‐dependent RNA polymerases.^8^ There are few published data on the pharmacokinetics of T‐705‐RTP. The reported *t*_1/2_ of T‐705‐RTP in human PBMCs is approximately 2 hours. The *t*_1/2_ of T‐705‐RTP in lung tissue in a murine model is 4.2 hours.^7^

Uncertainty exists around the use of favipiravir as a treatment for COVID‐19.^6^ Concerns include risk of teratogenicity, pill burden, and uncertain effectiveness against SARS‐CoV‐2.^9^, ^10^, ^11^, ^12^, ^13^ *In vitro* studies demonstrate inconsistent activity against SARS‐CoV‐2, with EC_50_ values ranging from 61.88 μM to > 500 μM, depending on experimental systems used.^11^, ^14^, ^15^, ^16^, ^17^ Effective antiviral activity has been shown in animal models.^18^, ^19^, ^20^ Clinical studies of oral favipiravir with pharmacokinetic (PK) characterization show significant inter‐individual variability in plasma concentrations^3^, ^21^ which in some cases diminish over time.^4^, ^22^, ^23^ Differences in PK exposures may depend on weight, critical illness, and ethnicity.^3^, ^22^

To maximize effectiveness, antiviral trough plasma concentrations should be maintained above the *in vitro* defined EC_90_ target.^17^, ^24^ The doses necessary to achieve this have been described by modeling based on published PK studies and likely concentrations of the active intracellular form (T‐705‐RTP).^25^

A novel intravenous (IV) formulation of favipiravir generates *C*_max_ levels 4‐fold higher than oral dosing in cynomolgus monkeys,^26^, ^27^ which may translate into sustained higher intracellular T‐705‐RTP concentrations and thus antiviral activity.^25^ A 2021 single ascending dose (300–2,400 mg), healthy volunteer study reported no serious adverse events (SAEs).^28^

We sought to assess safety and tolerability of multiple ascending doses of IV favipiravir in participants hospitalized with COVID‐19 and to recommend a safe dose for efficacy evaluation. Secondary objectives included PK characterization and virological and clinical outcomes.

## MATERIALS AND METHODS

### Study design, participants and ethics

This dose‐escalation phase Ib study was designed as an open‐label, randomized, controlled Bayesian adaptive trial in adult patients hospitalized with COVID‐19, recruited in the NIHR Liverpool Clinical Research Facility (UK). The Bayesian aspect of the dose‐escalation design allows efficiency in dose escalation decision making. Simulations show that between 32 and 40 participants are required across the dose finding and efficacy evaluation.^1^ The analysis plan allows for testing of up to 5 dose levels of IV Favipiravir in up to 5 cohorts of 6 patients each. The starting dose of favipiravir used in CST‐6 is lower than doses delivered in an oral formulation in extensive previous clinical trials. The choice of the highest dose level was informed by PK modeling studies.^25^ Justification of the starting dose including data from pre‐clinical and healthy volunteer studies is detailed in the CST‐6 protocol included in supplementary data (**S1**). Eligible participants were men and women aged ≥ 18 years with PCR‐confirmed SARS‐CoV‐2 infection within 7 days of randomization, within 14 days of symptom onset, with severity as defined by WHO Clinical Progression Scale grades 4–6 (this was expanded from the original criteria of grades 5–6 in v.3 of the protocol). Women of childbearing potential (WOCBP) and men who were sexually active with WOCBP were required to use a highly effective method of contraception. The following criteria excluded participation: pregnant or breastfeeding, stage 4 or greater chronic kidney disease, alanine aminotransferase (ALT) and/or aspartate aminotransferase (AST) > 5 times the upper limit of normal, anticipated transfer to another hospital within 72 hours, known allergy to any study medication, participation in any clinical trial of an investigational medicinal product (CTIMP) within 30 days. All participants, or legally acceptable representatives, provided written informed consent before enrolment. The study protocol was reviewed by the UK Medicines and Healthcare products Regulatory Agency (MHRA) (EudraCT 2020‐001860‐27) and West Midlands Edgbaston Research Ethics Committee (20/WM/0136).

### Randomization

Four sequential dosing tiers were defined *a priori* (600, 1,200, 1,800, 2,400 mg twice daily (b.i.d.) for 7 days or until discharge; a 300 mg tier was allowed in case of de‐escalation). Participants were recruited in cohorts of 6 and randomized to IV favipiravir or standard of care (SoC) in a 2:1 allocation ratio using permuted blocks with sentinel dosing in each cohort. Patients were registered on the trial database following consent; randomization was performed within the trial database when patients had completed screening and eligibility had been confirmed. The randomization sequence was generated and concealed via use of a web‐based system (MEDIDATA RAVE), specified and checked by Southampton CTU statisticians. Members of the trial team at site were blinded to allocation sequence and only aware of a participant's allocation when this was assigned on the trial database.

### Procedures

Inpatients at the Liverpool University Hospitals Foundation Trust with confirmed SARS‐CoV‐2 infection or symptoms consistent with COVID‐19 were screened for eligibility. Baseline demographic and medical information was collected. Participants randomized to treatment received an infusion of IV favipiravir over 1 hour, twice daily for 7 days, or until discharge if this occurred before day 7. Clinical information and tests for safety monitoring, virology and PK were collected at predefined points. Additional safety tests were carried out if indicated. A full schedule of procedures is included within the CST‐6 protocol (**S1**).

Dose escalation was guided by the Bayesian adaptive design of the AGILE platform.^1^ As with previous candidates^29^, ^30^ a dose toxicity model^31^ was used which estimates the probability of dose limiting toxicity (DLT) at days 8 and 29 in control groups and at each dosing level.^32^

**Figure 1:**
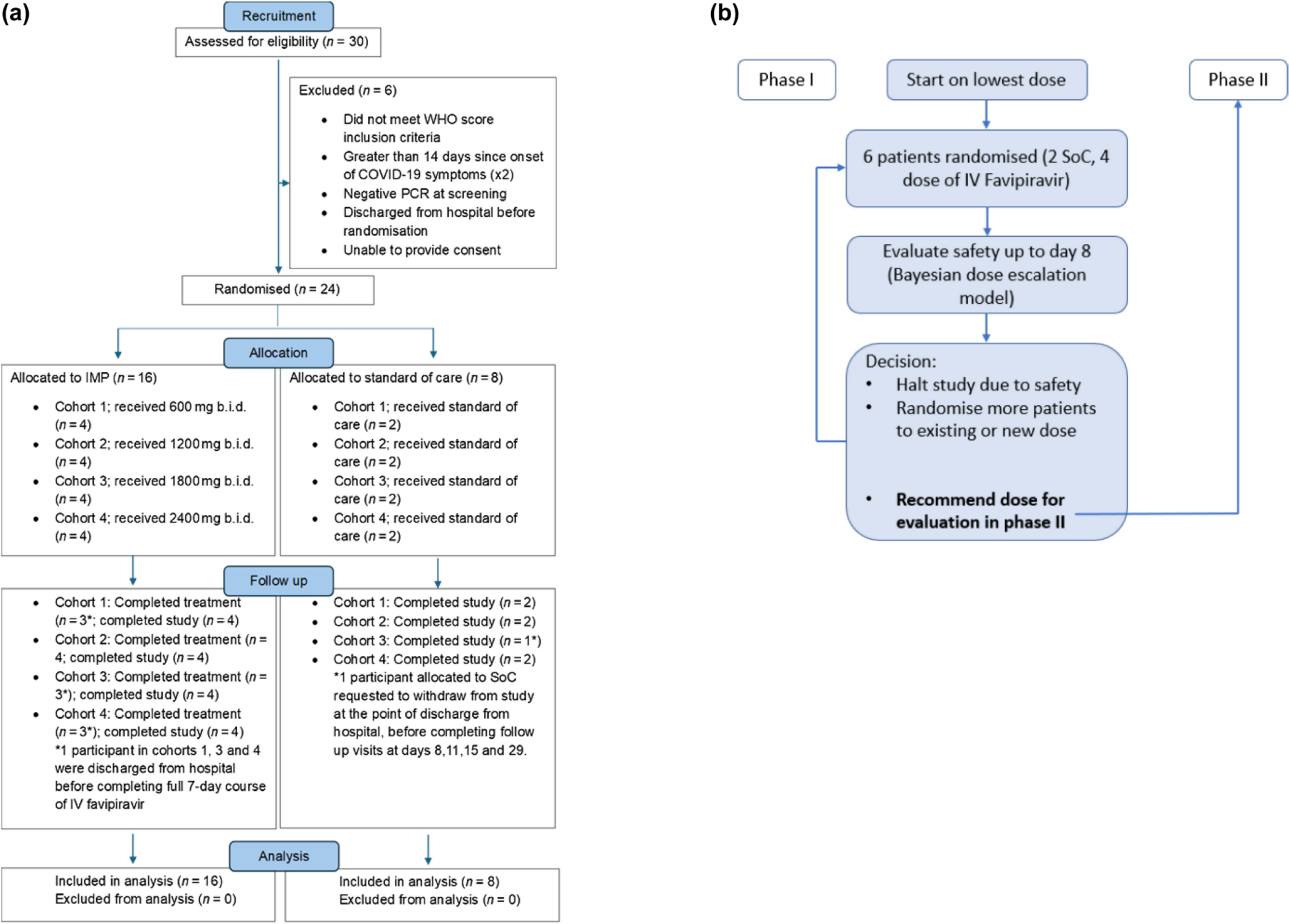
Trial design. (**a**) CST‐6 CONSORT diagram describing participant disposition and follow up. (**b**) Trial Schema for dose finding and efficacy evaluation, as described in the AGILE CST‐6 protocol (**S1**).

DLT was defined per the CST‐6 protocol as any AE ≥ grade 3 CTCAE v.5, possibly or probably related to the IMP. This definition was clarified in v.4 of the protocol to include an independent reviewer, blinded to allocation, to adjudicate causality of all AEs ≥ grade 3 with those that were deemed possibly or probably related to treatment included into the model as DLTs. A dose level was deemed unsafe if the chance that treatment was associated with a 30% increase in the probability of a DLT occurring by day 8 was 25% or greater. The Bayesian model recommended a course of action based on projected risk of DLT for the next dose level, with study cessation or dose de‐escalation recommended in the event of unacceptable toxicity. As per the master protocol,^1^ recruitment will cease if the probability that the risk of toxicity is at least 30% more than the control arm is 25% or more during phase I. On completion of each cohort, all available safety data were reviewed by the Safety Review Committee (SRC) who made the final decision on dose escalation, including ultimately a decision to recommend a safe dose for efficacy evaluation (**Figure** **1**). The role and make‐up of the SRC are defined in the CST‐6 protocol and SRC charter. The SRC is independent of sponsor and Fujifilm Toyama. The data management strategy including who was able to access interim results is outlined in the master protocol.^1^

### Outcomes

Primary outcomes were DLTs up to day 8 and all AEs and SAEs in all participants. Secondary outcomes included plasma PK parameters up to day 8, change from baseline over time in viral load up to day 29, clinical progression at days 15 and 29 as quantified by the WHO Clinical Progression Scale, mortality at days 15 and 29 and time from randomization to death (up to day 29).

### Pharmacokinetics

Plasma was sampled for favipiravir concentrations at days 1, 3, and 5. Two milliliters of venous blood was collected at 0–1 hours and 6–12 hours post‐completion of infusion. Median *T*_last_ values for each sampling timepoint are included with the results. On days 1 and 3, additional samples were taken pre‐dose (*C*_0_ and *C*_trough_, respectively) and at 2–4 hours post‐completion of infusion. Samples were cooled on wet ice, centrifuged (2,000 g for 10 min at 4°C), distributed to 2 cryovials (approx. 500 μL each), and transferred to a − 80°C freezer. Drug concentrations were measured using a validated LC–MS/MS assay^33^ at the Bioanalytical Facility (BAF) at the University of Liverpool.

Pharmacokinetic parameters were derived using non‐compartmental analysis with Phoenix 64 WinNonlin (version 8.3).

### Pharmacokinetic modeling

Plasma concentrations were modeled as a one‐compartment^3^, ^25^ IV infusion population pharmacokinetic (pop‐PK) model with first order clearance using nlmixr2 (version 3.0.2).^34^ Measurements below LLQ were labeled as 500 ng/mL with the exception of day 1 pre‐dose measurements which were labeled as zero. A nonlinear mixed effects model was used to estimate the clearance and volume of distribution, scaled by bodyweight according to:

$$\mathrm{Cl}={\mathrm{Cl}}_{70\mathrm{kg}}\;{\left(\frac{\mathrm{BW}}{70}\right)}^{0.75}$$

$$V_{d}={V_{d}}_{70\mathrm{kg}}\;{\left(\frac{\mathrm{BW}}{70}\right)}^{1}$$

${\mathrm{Cl}}_{70\mathrm{kg}}$ and ${V_{d}}_{70\mathrm{kg}}$ denote the clearance and volume of distribution for an individual with a bodyweight of 70 kg respectively and BW is the bodyweight of the individual. A proportional error on plasma concentrations was also included. Prediction‐corrected visual predictive check (PC‐VPC) was produced using the built‐in VPC function in nlmixr2.

For simulations, 2,000 virtual participants were generated using Monte Carlo sampling of the bodyweight, clearance and volume of distribution, based on the probability distribution of fitted values. Body weights were sampled from a truncated Gaussian distribution with the mean, standard deviation, lower and upper bounds equal to those of the trial participants.

### Statistical analysis

All analyses are reported according to CONSORT 2010 and ICH E9 guidelines on Statistical Principles in Clinical Trials. All participants were included in both the evaluable population and the safety population for analysis. The primary endpoint of DLTs up to 8 days after first dose was modeled using a Bayesian dose–toxicity model based on Mozgunov *et al*.^31^, ^32^ The structure of the dose toxicity model is

$$\varphi \left(\tilde{d_{j}},\theta_{1},\theta_{2}\right)=\frac{\exp\left(\theta_{1}+\theta_{2}\tilde{d_{j}}\right)}{1+\exp\left(\theta_{1}+\theta_{2}\tilde{d_{j}}\right)}$$

where $\tilde{d_{j}}$ are the standardized levels obtained through prior estimates of the DLT probabilities ${\hat{p_{j}}}^{\left(0\right)}$:

$$\tilde{d_{j}}=\frac{\text{logit}\left({\hat{p_{j}}}^{\left(0\right)}\right)-{\hat{\theta_{1}}}^{\left(0\right)}}{{\hat{\theta_{2}}}^{\left(0\right)}}$$

where ${\hat{\theta_{1}}}^{\left(0\right)}$, ${\hat{\theta_{2}}}^{\left(0\right)}$ are prior point estimates of the model parameters, with $\tilde{d_{j}}$= 0.

The relationship between dose and toxicity was modeled using a two‐parameter logistic model, where information can be shared across doses; the DLT rate in controls informs estimates for the active doses. The prior distributions for this model were calibrated to maximize the proportion of correct selection under a range of dose–toxicity scenarios where each dose considered was the optimum one (details included in **S4**). The toxicity risk in controls was *a priori* assumed to be 10%.

The model was updated after each cohort, with the final model presented as estimated DLT rates for each dose, alongside equal‐tail 95% credible intervals. We report estimated additional toxicity above controls, the probability that the DLT rate falls within 15–25% additional toxicity over controls (a predetermined acceptable range) and the probability of at least 30% additional toxicity over controls (deemed unacceptable).

Baseline demographics are summarized using descriptive statistics. Clinical endpoints are summarized at days 8, 15, and 29. The sample size was flexible to adapt to cumulative safety data. Simulations were performed to assess model operating characteristics and to calibrate priors, assuming five doses (plus controls), with cohorts of size six capped at a total of 42 participants.

Statistical analysis was undertaken in SAS version 9.4, STATA version 16 and R version 3.6.0.

## RESULTS

30 potential participants were identified for screening, 6 were excluded (WHO score, > 14 days COVID‐19 symptoms [x2], negative PCR, discharged before randomization, unable to provide consent; described in **Figure** **1**). Baseline characteristics are described in **Table** **1** and were similar across all groups. Four sequential dose cohorts (600, 1,200, 1,800 and 2,400 mg b.i.d.) of six participants each were randomized and received doses of IV favipiravir or SoC in the period between September 10, 2022 and November 1, 2023. All participants randomized to treatment received at least 1 dose and 13 completed the full treatment course. Three participants (1 in each of the 600, 1,800 and 2,400 mg cohorts) did not complete the full course, due to discharge from hospital. One participant allocated to SoC requested to withdraw, prior to planned follow up at days 8, 11, 15 and 29.

**Table 1:** Demographic and baseline characteristics—evaluable population

Demographic characteristics	Favipiravir	Favipiravir	Favipiravir	Favipiravir	Favipiravir	Standard of care	Total
	600 mg (*N* = 4)	1,200 mg (*N* = 4)	1,800 mg (*N* = 4)	2,400 mg (*N* = 4)	Total (*N* = 16)	(*N* = 8)	(*N* = 24)
Age at consent (years)							
Median (Q1, Q3)	82.5 (75.5, 90.5)	76.5 (64.5, 84.0)	66.0 (56.0, 79.0)	76.5 (72.5, 81.5)	76.5 (70.5, 86.0)	73.5 (65.5, 77.0)	74.0 (69.0, 81.0)
Min, Max	74.0, 93.0	57.0, 87.0	52.0, 86.0	69.0, 86.0	52.0, 93.0	57.0, 81.0	52.0, 93.0
Gender, *n* (%)							
Male	2.0 (50.0)	2.0 (50.0)	4.0 (100.0)	1.0 (25.0)	9.0 (56.3)	5.0 (62.5)	14.0 (58.3)
Female	2.0 (50.0)	2.0 (50.0)	0.0 (0.0)	3.0 (75.0)	7.0 (43.8)	3.0 (37.5)	10.0 (41.7)
Weight (kg)							
Median (Q1, Q3)	68.3 (60.5, 78.1)	70.80 (54.7, 96.3)	85.4 (75.1, 107.0)	87.1 (63.8, 99.5)	78.6 (60.5, 93.9)	78.45 (67.3, 102.9)	78.45 (65.2, 99.3)
Min, Max	57.2, 83.3	53.4, 107.0	68.4, 125.0	52.1, 100.1	52.1, 125.0	55.0, 117.4	52.1, 125.0
BMI (kg/m^2^)							
Median (Q1, Q3)	25.8 (23.5, 27.2)	27.0 (23.7, 32.3)	28.2 (26.8, 35.9)	30.0 (24.6, 36.8)	27.8 (24.5, 30.2)	25.5 (23.8, 32.9)	27.2 (24.5, 31.0)
Min, Max	22.3, 27.5	23.1, 34.9	25.4, 43.4	19.1, 42.7	19.1, 43.4	21.8, 36.2	19.1, 43.4
Ethnicity, *n* (%)							
White—English, Welsh, Scottish, Northern Irish	4.0 (100.0)	3.0 (75.0)	4.0 (100.0)	4.0 (100.0)	15.0 (93.8)	8.0 (100.0)	23.0 (95.8)
White ‐ other	0.0 (0.0)	1.0 (25.0)	0.0 (0.0)	0.0 (0.0)	1.0 (6.3)	0.0 (0.0)	1.0 (4.2)
First recorded COVID‐19 infection, *n* (%)							
Yes	4.0 (100.0)	3.0 (75.0)	3.0 (75.0)	3.0 (75.0)	13.0 (81.3)	7.0 (87.5)	20.0 (83.3)
Received vaccine for COVID‐19, *n (%)*							
Yes	3.0 (75.0)	4.0 (100.0)	4.0 (100.0)	3.0 (75.0)	14.0 (87.5)	6.0 (75.0)	20.0 (83.3)
WHO score (baseline)							
Median, range	5.0 (5.0–5.0)	4.0 (4.0–5.0)	5.0 (4.0–5.0)	5.0 (4.0–5.0)	5.0 (4.0–5.0)	5.0 (4.0–5.0)	5.0 (4.0–5.0)
Number of listed co‐morbidities							
Median, range	11.0 (9.0–13)	11.5 (6.0–16.0)	12.0 (5.0–19.0)	11.0 (10.0–13.0)	11.0 (5.0–19.0)	10.5 (5.0–19.0)	11.0 (5.0–19.0)

*N*, number of participants in the evaluable population.

BMI, body mass index; WHO score, World Health Organization clinical progression score.

### Primary analysis

**Figure 2:**
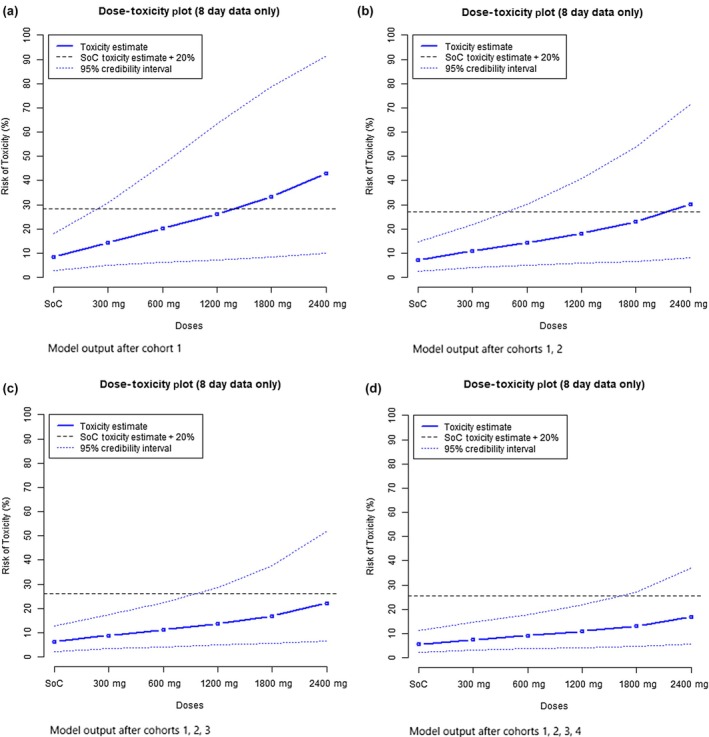
Bayesian model estimates of dose toxicity. Bayesian model output plots, updated after each cohort and presented as estimated DLT rates for each dose, alongside equal‐tail 95% credible intervals. Plots remain identical after all data up to day 29 are included.

No DLTs were observed in any participant. Doses were escalated as planned to a maximum of 2,400 mg b.i.d. Bayesian model DLT point estimates with 95% credible intervals, calculated after each cohort, are shown in **Figure** **2**. The maximum dose level had an estimated DLT rate of 16.8% at day 8, with an estimated 11.2% additional toxicity over controls. The probability that toxicity fell within the predetermined target range of 15–25% additional toxicity over controls was 18.3%, which was the highest probability across all doses. The probability of ≥ 30% additional toxicity over controls was 2.7%. These values remained the same when data up to day 29 were included. These findings support the use of this dose in future phase II studies.

AEs across all cohorts are detailed in **Table** **2**. At least 1 AE was experienced by all participants allocated to favipiravir. Of those allocated to SoC, 5 of 8 (62.5%) experienced at least 1 AE. AEs deemed related to favipiravir were experienced by 1 of 4 (25%), 3 of 4 (75%), 2 of 4 (50%) and 4 of 4 (100%) in the 600, 1,200, 1,800 and 2,400 mg b.i.d. cohorts respectively. All 10 related AEs were asymptomatic hyperuricemia. The most common AEs by category were metabolism and nutrition disorders, infections and infestations, and gastrointestinal disorders. AEs with severity ≥ grade 3 were experienced by 2 of 4 (50%), 2 of 4 (50%), 2 of 4 (50%) and 1 of 4 (25%) in the 600, 1,200, 1,800 and 2,400 mg b.i.d. cohorts respectively, and 4 of 8 (50%) of those allocated to SoC.

**Table 2:** Adverse events by system organ class and CTCAE term—evaluable population

System organ class	Favipiravir	Favipiravir	Favipiravir	Favipiravir	Favipiravir	Standard of care	Total
	600 mg (*N* = 4)	1,200 mg (*N* = 4)	1,800 mg (*N* = 4)	2,400 mg (*N* = 4)	Total (*N* = 16)	(*N* = 8)	(*N* = 24)
	*n* (%)	*n* (%)	*n* (%)	*n* (%)	*n* (%)	*n* (%)	*n* (%)
Participants with any system organ class	4.0 (100.0)	4.0 (100.0)	4.0 (100.0)	4.0 (100.0)	16.0 (100.0)	5.0 (62.5)	21.0 (87.5)
Blood and lymphatic system disorders	1.0 (25.0)	0.0 (0.0)	0.0 (0.0)	0.0 (0.0)	1.0 (6.3)	0.0 (0.0)	1.0 (4.2)
Cardiac disorders	0.0 (0.0)	0.0 (0.0)	0.0 (0.0)	0.0 (0.0)	0.0 (0.0)	1.0 (12.5)	1.0 (4.2)
Eye disorders	0.0 (0.0)	0.0 (0.0)	1.0 (25.0)	0.0 (0.0)	1.0 (6.3)	0.0 (0.0)	1.0 (4.2)
Gastrointestinal disorders	2.0 (50.0)	0.0 (0.0)	2.0 (50.0)	2.0 (50.0)	6.0 (37.5)	1.0 (12.5)	7.0 (29.2)
General disorders and administration site conditions	3.0 (75.0)	1.0 (25.0)	0.0 (0.0)	1.0 (25.0)	5.0 (31.3)	1.0 (12.5)	6.0 (25.0)
Hepatobiliary disorders	0.0 (0.0)	1.0 (25.0)	0.0 (0.0)	0.0 (0.0)	1.0 (6.3)	0.0 (0.0)	1.0 (4.2)
Immune system disorders	0.0 (0.0)	0.0 (0.0)	0.0 (0.0)	1.0 (25.0)	1.0 (6.3)	0.0 (0.0)	1.0 (4.2)
Infections and infestations	1.0 (25.0)	1.0 (25.0)	1.0 (25.0)	3.0 (75.0)	6.0 (37.5)	3.0 (37.5)	9.0 (37.5)
Injury, poisoning and procedural complications	0.0 (0.0)	0.0 (0.0)	1.0 (25.0)	1.0 (25.0)	2.0 (12.5)	0.0 (0.0)	2.0 (8.3)
Investigations	0.0 (0.0)	1.0 (25.0)	1.0 (25.0)	0.0 (0.0)	2.0 (12.5)	1.0 (12.5)	3.0 (12.5)
Metabolism and nutrition disorders	3.0 (75.0)	4.0 (100.0)	2.0 (50.0)	4.0 (100.0)	13.0 (81.3)	4.0 (50.0)	17.0 (70.8)
Musculoskeletal and connective tissue disorders	0.0 (0.0)	0.0 (0.0)	1.0 (25.0)	1.0 (25.0)	2.0 (12.5)	1.0 (12.5)	3.0 (12.5)
Nervous system disorders	0.0 (0.0)	1.0 (25.0)	0.0 (0.0)	3.0 (75.0)	4.0 (25.0)	0.0 (0.0)	4.0 (16.7)
Psychiatric disorders	2.0 (50.0)	0.0 (0.0)	0.0 (0.0)	0.0 (0.0)	2.0 (12.5)	1.0 (12.5)	3.0 (12.5)
Renal and urinary disorders	1.0 (25.0)	1.0 (25.0)	0.0 (0.0)	0.0 (0.0)	2.0 (12.5)	1.0 (12.5)	3.0 (12.5)
Respiratory, thoracic and mediastinal disorders	1.0 (25.0)	1.0 (25.0)	1.0 (25.0)	2.0 (50.0)	5.0 (31.3)	2.0 (25.0)	7.0 (29.2)
Skin and subcutaneous tissue disorders	1.0 (25.0)	1.0 (25.0)	0.0 (0.0)	0.0 (0.0)	2.0 (12.5)	0.0 (0.0)	2.0 (8.3)
Vascular disorders	0.0 (0.0)	1.0 (25.0)	0.0 (0.0)	0.0 (0.0)	1.0 (6.3)	0.0 (0.0)	1.0 (4.2)

*N*, number of participants in the evaluable population. Percentages are based on the total number of participants in each cohort.

SAEs were experienced by 1 of 4 (25%), 1 of 4 (25%), 2 of 4 (50%) and 0 of 4 (0%) in the 600, 1,200, 1,800 and 2,400 mg b.i.d. cohorts respectively, and 1 of 8 (12.5%) of those allocated to SoC. No SAEs were deemed related to favipiravir.

### Analysis of secondary endpoints

#### Clinical scores

There was no difference in the median WHO score between the treatment and SoC groups at baseline, day 8, day 15, and day 29. The 2,400 mg b.i.d. cohort had lower median WHO scores than the SoC group at days 8 and 15. Small numbers and clinical heterogeneity limit interpretation of these differences.

There were no deaths in all participants up to the end of the study.

#### Pharmacokinetics

**Figure 3:**
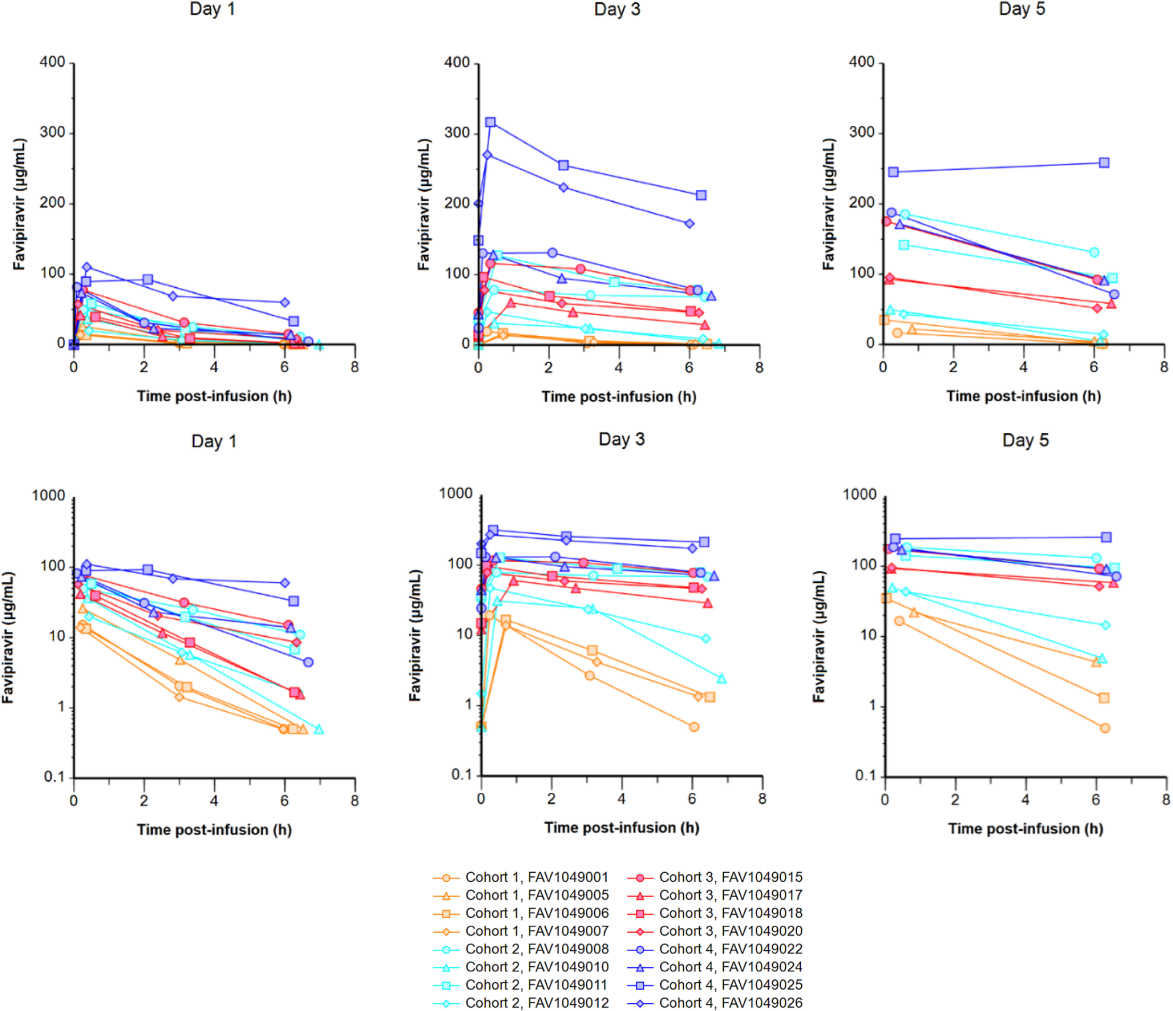
Favipiravir plasma pharmacokinetic profile. Favipiravir concentrations over actual time post‐infusion stratified by study day in hospitalized patients with COVID‐19 receiving intravenous favipiravir 600 mg twice daily over 1 hour (cohort 1: orange closed markers, solid lines), 1,200 mg twice daily over 1 hour (cohort 2: cyan closed markers, solid lines), 1,800 mg twice daily over 1 hour (cohort 3: red closed markers, solid lines) and 2,400 mg over 1 hour (cohort 4: blue closed markers, solid lines) on a linear (top) and log‐linear (bottom) scale (cohort 1: *n* = 4 study day 1, *n* = 3 study days 3 and 5; cohort 2: *n* = 4 study days 1, 3, and 5; cohort 3 and 4: *n* = 4, study days 1 and 3, *n* = 3 study day 5).

**Figure 4:**
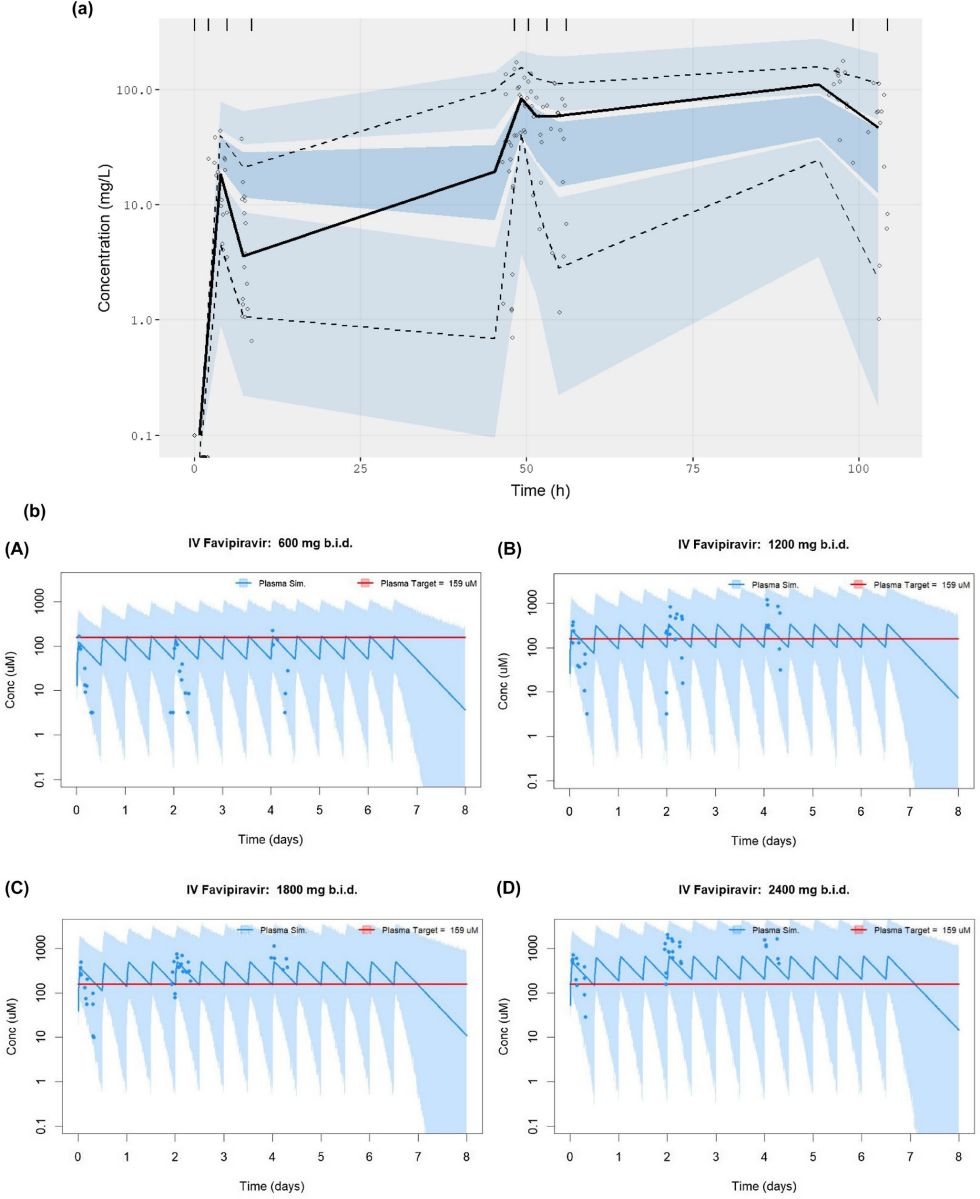
Population‐pharmacokinetic modeling. (**a**) A Prediction‐Corrected Visual Predictive Check of the fitted 1‐compartment IV infusion model. The solid black line represents the median and the dashed lines represent the 5th and 95th percentile of the data. The dark blue and light blue shaded areas represent the 90% prediction interval (5th to 95 percentiles) of the simulated median, 5th and 95th percentiles based on 2,000 simulated individuals. Binning for prediction intervals matched plasma measurement windows as indicated by vertical tick marks. (**b**) Simulations of favipiravir plasma concentrations following twice‐daily, 1 hour IV infusions of favipiravir at 600 mg (**A**), 1,200 mg (**B**), 1,800 mg (**C**) and 2,400 mg (**D**) b.i.d. The blue line and blue shaded area represent the median and the 90% prediction interval of 2,000 simulations respectively. Observed data is represented by blue circles. The red line indicates the *in vitro* EC_90_ of favipiravir against SARS‐CoV‐2 in Vero‐E6 cells.^17^

Significant variability in PK parameters between individuals within each cohort is the preeminent characteristic of these data, represented in **Figure** **3**. Plasma favipiravir was below the lower limit of quantification (LLQ; < 1 μg/mL) in all day 1 pre‐dose samples, in all day 1 *C*_last_ (6–12 hours post completion of infusion, median *T*_last_ 6.1 hours) samples in the 600 mg cohort, in all day 3 pre‐dose samples and in 1 of 4 (25%) day 3 *C*_last_ samples in the 600 mg cohort. In all other samples favipiravir was quantifiable, summary data are described in **Table** **3**. Plasma favipiravir concentrations demonstrated accumulation between day 1 and day 3 in all cohorts. At day 3, median favipiravir exposures (AUC_0‐last_) for the 600 mg (*N* = 3), 1,200 mg (*N* = 4), 1,800 mg (*N* = 4) and 2,400 mg (*N* = 4) cohorts were 38.54, 307.48, 383.03 and 1,004.18 μg.h/mL respectively, with corresponding median *C*_max_ values of 16.80, 62.95, 87.51 and 200.99 μg/mL. Median day 3 *C*_trough_ values (pre‐dose on day 3) were < 1, 17.25, 19.46 and 96.49 μg/mL. *T*_max_, the time after completion of a 1 hour infusion to maximum concentration, was <1 hour in the 600, 1,200 and 1,800 mg cohorts. In the 2,400 mg cohort *T*_max_ ranged from 0.08 to 6.28 hours. One plasma sample in the 600 mg cohort on day 3 was excluded from all analyses due to a sampling error. A 1‐compartment pop‐PK model was produced using the data from this study (**Figure** **4**, S3). The estimated clearance and volume of distribution (supplementary data, **S2**) conformed with previous studies of oral favipiravir.^3^ Plasma concentrations appear to show a dose dependent nonlinear PK however fitting a dose dependent clearance was not possible due to insufficient data. Dose‐stratified simulations show a mean *C*_trough_ exceeds the plasma target concentration of 159 μM at 2,400 mg b.i.d. This suggests that a dose between 1,800 and 2,400 mg b.i.d. may be sufficient to achieve pre‐specified PK targets for SARS‐CoV‐2.^24^

**Table 3:** Summary of favipiravir plasma pharmacokinetic parameters in hospitalized patients receiving intravenous favipiravir

Day	Dose (mg, twice daily)	*T*_max_ (h)	*C*_0/trough_ (μg/mL)^a^	*C*_max_ (μg/mL)	*T*_last_ (h)	*C*_last_ (μg/mL)	AUC_0‐last_ (h*μg/mL)
Day 1	600, *N* = 4	0.25 (0.18–0.35)	b	14.59 (13.34–26.03)	6.10 (5.95–6.52)	0.50 (0.50–0.50)^c^	28.74 (25.73–55.39)
	1,200, *N* = 4	0.41 (0.27–0.50)	b	43.04 (19.83–58.21)	6.41 (6.28–6.97)	4.24 (0.50^c^‐11.04)	119.90 (51.45–177.80)
	1,800, *N* = 4	0.22 (0.12–0.62)	b	49.55 (39.40–77.75)	6.30 (6.10–6.43)	5.11 (1.56–15.12)	120.40 (91.68–236.20)
	2,400, *N* = 4	0.29 (0.08–2.10)	b	87.17 (73.44–110.3)	6.21 (6.00–6.67)	23.57 (4.47–59.86)	315.00 (179.00–444.50)
Day 3	600, *N* = 3	0.70 (0.23–0.70)	0.50 (0.50–0.50)^c^	16.80 (13.83–19.42)	6.17 (6.05–6.50)	1.34 (0.50^c^‐1.35)	38.54 (36.31–46.68)
	1,200, *N* = 4	0.44 (0.25–0.53)	17.25 (0.50–37.10)	62.95 (31.03–127.40)	6.41 (6.20–6.83)	38.73 (2.45–78.07)	307.50 (130.20–601.60)
	1,800, *N* = 4	0.25 (0.13–0.92)	19.46 (12.15–46.17)	87.51 (60.34–116.20)	6.15 (6.02–6.43)	47.00 (28.93–77.73)	383.00 (270.30–605.10)
	2,400, *N* = 4	0.38 (0.25–2.10)	96.49 (24.46–201.40)	201.00 (128.50–317.00)	6.28 (6.00–6.62)	125.50 (70.50–213.10)	1,004.20 (606.00–1,592.30)
Day 5	600, *N* = 3	0.40 (0.03–0.82)	d	22.10 (16.69–35.01)	6.16 (6.00–6.25)	1.34 (0.50^c^‐4.35)	e
	1,200, *N* = 4	0.58 (0.20–0.62)	d	95.85 (43.25–185.50)	6.24 (6.00–6.52)	54.52 (4.92–131.20)	e
	1,800, *N* = 3	0.17 (0.08–0.18)	d	95.41 (92.89–175.10)	6.22 (6.08–6.48)	58.39 (51.50–92.31)	e
	2,400, *N* = 3	0.47 (0.23–6.28)	d	187.90 (171.40–258.50)	6.38 (6.28–6.57)	91.03 (71.52–258.50)	e

All values represented as median (range). *N*, number of participants in the evaluable population.

AUC_0‐last_: area under the concentration‐time curve from pre‐dose to last measured sample; *C*_0_; pre‐dose concentration, day 1; *C*_last_: concentration of the last measured timepoint; *C*_max_: maximum measured concentration; *C*_trough_; pre‐dose concentration, day 3; *T*_last_: time after completion of infusion of last measured concentration; *T*_max_: time after completion of infusion of maximum concentration.

^a^ Sample prior to first dose of the day; Day 1 = *C*_0_, Day 3 = *C*_trough_.

^b^ Sample taken prior to first dose, favipiravir undetectable.

^c^ Below LLQ and imputed as LLQ/2.

^d^ As per protocol, no pre‐dose sample taken on day 5.

^e^ Insufficient data points on day 5 to calculate AUC.

#### Virology

Detailed pharmacodynamic analysis is beyond the scope of this study. There was no significant difference in change from baseline viral load between the treatment and SoC groups. There was heterogeneity in individual drug exposure, concomitant antiviral medications, and baseline viral load.

## DISCUSSION

We describe the first use of IV favipiravir in hospitalized patients with SARS‐CoV‐2 infection and include characterization of its safety, tolerability and pharmacokinetic profile. We demonstrate that IV favipiravir is safe and well tolerated at doses up to 2,400 mg b.i.d., in the context of the limited numbers of a phase Ib trial. AEs were reported in all cohorts, reflecting symptomatic COVID‐19 and co‐morbidity associated with a realistic patient population, characterized by co‐morbidity and frailty to an unusual degree for early phase studies. A higher number of AEs was reported in participants allocated to treatment over SoC, however there was no dose dependent association. Hyperuricemia was seen in 14 participants (10/16 allocated to favipiravir, 4/4 at the highest dose level; 4/8 allocated to SoC). In all cases this resolved following completion of treatment.

All AEs of severity ≥ grade 3 CTCAE v.5 were reviewed by a third‐party independent assessor with access to all clinical data but blinded to treatment allocation; those deemed possibly or probably related to favipiravir were classified as DLTs and included in the model. In the final analysis the probability of > 30% excess toxicity over controls at 2,400 mg as estimated by the Bayesian model was 2.7%.

As with previous larger studies of oral favipiravir with PK characterization,^21^ we observed marked inter‐individual variability, supporting the idea of complex metabolism. The degree to which individual factors, including weight, sex, genetic variability, ethnicity and clinical status, contribute to this variability remains incompletely understood.^3^, ^22^, ^35^ Our general approach in setting pre‐specified PK targets in COVID‐19 trials within the AGILE trial platform was that antiviral efficacy typically requires sustained exposure above the EC_90_ as a minimum. Wide inter‐individual variability in PK parameters means making dose recommendations based on meeting pre‐specified PK targets is challenging.

We considered that IV dosing may lead to transiently higher plasma concentrations, resulting in higher intracellular T‐705‐RTP levels,^25^ and thus greater activity and utility in more unwell patients. We did not use a loading dose, as our final target doses were in the range used as loading doses in other studies, and to investigate a previously observed decline in plasma favipiravir concentrations between days 3 and 5.

A significant fall in plasma favipiravir concentration has been observed after several days of oral dosing, following a loading dose and in critically unwell patients.^4^, ^23^, ^36^ In our study accumulation in plasma was seen at days 3 and 5, suggesting differences in accumulation following a loading dose or potentially by route of administration. Our final cohort used higher sustained doses of favipiravir than previously published studies. In the 2,400 mg b.i.d. cohort, plasma favipiravir day 3 trough levels were close to or above the pre‐specified target EC_90_ (24.9 μg/mL; 159 μM). Population‐PK modeling suggests that doses of between 1,800 and 2,400 mg b.i.d. may achieve pre‐specified target concentrations (**Figure** **4**, S3). Our data suggest that studies of oral favipiravir in COVID‐19 using lower doses are unlikely to have achieved sustained plasma favipiravir concentrations above the target EC_90_. Indeed, a wide range of EC_50_ values have been reported and more recently both published and unpublished work suggest an EC_90_ closer to 500 μM. It has been demonstrated that the levels of active metabolite T‐705‐RTP generated in different *in vitro* systems can vary significantly.^37^, ^38^ Selection of PK targets based on pre‐clinical data is challenging due to this variability and assumptions around T‐705‐RTP formation. We acknowledge the uncertainty in translating *in vitro* antiviral potential in to *in vivo* clinical effect and do not assert that meeting these PK targets necessarily translates to clinical efficacy. Contrastingly, *in vivo* animal infection models have demonstrated potent antiviral effectiveness at considerably lower *C*_trough_ levels (mean *C*_trough_ 29.9 μg/mL at the highest dose level) than those achieved in CST‐6.^18^, ^20^

Interpretation of virological and clinical outcomes is limited by small numbers and heterogeneity of baseline data; this phase I study was not powered to assess efficacy of IV favipiravir against SARS‐CoV‐2.

IV favipiravir was well tolerated at doses up to 2,400 mg b.i.d. that reached pre‐specified PK targets in a study group with frailty and complex health profiles. Our data support the safety of favipiravir at these doses in future phase II studies.

## FUNDING

The AGILE trial is an academic, non‐commercial trial sponsored by the University of Liverpool. This work is supported by the Medical Research Council (grant numbers MR/V028391/1, MR/W005611/1); the Wellcome Trust (grant number 221590/Z/20/Z); the US Food and Drug Administration (75F40120C00085) and the National Institute for Health and Care Research (award 200907). P.M. is supported by the National Institute for Health and Care Research through the NIHR Advanced Fellowship (NIHR300576). P.M. and T.J. are supported by the Medical Research Council (MC_UU_00040/03). G.G. receives funding for SCTU staff from the National Institute for Health Research Southampton Biomedical Research Centre. The article reflects the views of the authors and does not represent the views or policies of the FDA, the NIHR or the Department of Health and Social Care.

## CONFLICTS OF INTEREST

S.H.K. has received research funding from ViiV Healthcare, Gilead Sciences, Pfizer, and Merck Sharp & Dohme for the Liverpool HIV Drug Interactions program and for unrelated clinical studies. G.G. has received funding from Janssen‐Cilag, AstraZeneca, Novartis, Astex, Roche, Heartflow, Celldex, BMS, BioNTech, MSD, IntraOp, Synairgen, Boehringer Ingelheim, Blood Cancer UK, Cancer Research UK, the NIHR, Asthma and Lung UK, UKRI, Wellcome Trust, NHS England, Unitaid, Imugene, and GSK for unrelated academic clinical trials and program funding and honoraria/consulting fees from AstraZeneca and AbbVie. A.O. is a director of Tandem Nano Ltd and co‐inventor of drug delivery patents. A.O. has been a co‐investigator on funding received by the University of Liverpool or Tandem Nano Ltd. from ViiV Healthcare, Bicycle Therapeutics, and Gilead Sciences and has received personal fees from Gilead, Shionogi, and Assembly Biosciences. All other authors declared no competing interests for this work.

## AUTHOR CONTRIBUTIONS

T.R., S.H.K., T.E.F. and R.F. wrote the manuscript; S.H.K., G.G., R.F., T.E.F., T.J., T.R., P.M., D.G.L., A.O., M.J. designed the research; T.E.F., R.L., R.F., L.W., T.R., K.D., M.I., Y.E., H.R., M.T., J.G., J.C., O.O. performed the research; S.H.K., G.G., G.S., W.W., H.P., A.O., T.E.F., R.F., T.R., L.D., E.C., L.J.E., V.S., W.G., C.H., C.K., K.B., J.A.H. analyzed the data.

## DATA SHARING STATEMENT

The AGILE Trial Steering Committee will consider all reasonable requests by health‐care providers, investigators, and researchers to provide anonymized data to address specific scientific or clinical objectives. The AGILE investigators are committed to reviewing requests from researchers for access to clinical trial protocols, de‐identified patient‐level clinical trial data, and study‐level clinical trial data. Data will be assigned a DOI through deposition in the University of Liverpool Research Data Catalogue (rdm@liverpool.ac.uk) and shared under a Data Transfer agreement (or equivalent e.g. as part of a research collaboration agreement or confidentiality disclosure agreement).

## Supporting information


**Data S1**. AGILE CST‐6 protocol

**Data S2**. Summary of parameter estimates for favipiravir 1‐compartment IV infusion POP‐PK model fitting

**Data S3**. PK parameters generated from popPK model

**Data S4**. Prior Parameters for the Randomised Bayesian Dose‐Escalation Model for CST‐6

**Data S5**. Reporting checklist for dose escalation or de‐escalation trial
