# Supplementary material for: Optimal Dose and Safety of Intravenous Favipiravir in Hospitalized Patients With COVID‐19: A Dose‐Escalating, Randomized Controlled Phase Ib Study
Source: Clin Pharmacol Ther. 2026 Mar 18;119(6):1650–61. doi: 10.1002/cpt.70261 (PMC13156351; doi:10.1002/cpt.70261)
Supplement: Supplementary file 1 — Data S1. AGILE CST‐6 protocol [file CPT-119-1650-s002.pdf]

AGILE PLATFORM PROTOCOL

CANDIDATE SPECIFIC TRIAL PROTOCOL 6 (CST-6): FAVIPIRAVIR

A Randomized, Multicentre, Seamless, Adaptive, Phase I/II Platform Study to Determine the Phase II dose and to Evaluate the Safety and Efficacy of intravenous Favipiravir for the Treatment of COVID-19

Master Protocol Title: AGILE: Seamless Phase I/II Platform for the Rapid Evaluation of Candidates for COVID-19 treatment

CST-6 version 4.0 13 Jan 2023

SPONSOR: University of Liverpool
COORDINATING CENTRE: Southampton Clinical Trials Unit

**Candidate Specific Trial Protocol authorised by:**			
**Name:** Prof Saye Khoo		**Role:** AGILE Chief Investigator	
**Signature:** Saye Khoo<br>Saye Khoo (Mar 16, 2023 14:26 GMT)		**Date:** Mar 16, 2023	
**Name:** Dr Tom Fletcher		**Role:** Candidate Chief Investigator	
**Signature:** T E Fletcher<br>T E Fletcher (Mar 16, 2023 14:38 GMT)		**Date:** Mar 16, 2023	
**Name:** Prof Gareth Griffiths		**Role:** Director of SCTU	
**Signature:** G Griffiths<br>G Griffiths (Mar 23, 2023 20:15 GMT)		**Date:** Mar 23, 2023	
**Name:** Karen Wilding		**Role:** On behalf of Sponsor	
**Signature:** Karen Wilding<br>Karen Wilding (Mar 24, 2023 07:46 GMT)		**Date:** Mar 24, 2023	

DEFINITIONS

AGILE Chief Investigator (CI): The Chief Investigator of the AGILE platform

Candidate Chief Investigator (CCI): The Chief Investigator for the candidate specific protocol within the AGILE platform

TABLE OF CONTENTS

LIST OF ABBREVIATIONS SPECIFIC TO THIS CANDIDATE SPECIFIC TRIAL (CST) PROTOCOL 5
1 CANDIDATE SPECIFIC PROTOCOL SUMMARY 6
1.1 CANDIDATE SPECIFIC SYNOPSIS 6
1.2 CANDIDATE SPECIFIC TRIAL SCHEMA 10
1.3 SCHEDULE OF OBSERVATIONS AND PROCEDURES (PHASE I AND II) 11
2 INTRODUCTION 13
2.1 BACKGROUND AND RATIONALE 13
2.2 RISK/BENEFITS FOR TREATMENT ARM / IMP 14
3 CANDIDATE SPECIFIC OBJECTIVES AND ENDPOINTS 21
3.1 PHASE I OBJECTIVES & ENDPOINTS 21
3.2 PHASE II OBJECTIVES & ENDPOINTS 22
4 TRIAL DESIGN 23
4.1 CANDIDATE SPECIFIC DESIGN 23
4.2 TRIAL PHASES 23
4.3 JUSTIFICATION FOR DOSE 24
4.4 DEFINITION OF END OF TRIAL 25
5 SELECTION AND ENROLMENT OF PATIENTS 25
5.1 INCLUSION CRITERIA 26
5.2 EXCLUSION CRITERIA 26
5.3 SCREEN FAILURES 27
5.4 CONTRACEPTION 27
5.5 REGISTRATION / RANDOMISATION PROCEDURES 27
6 TREATMENTS 27
6.1 TREATMENT SCHEDULE 27
6.2 IMP SUPPLY 28
6.3 PRODUCT HANDLING, STORAGE AND STABILITY 28
6.4 PREPARATION AND ADMINISTRATION 28
6.5 ACCOUNTABILITY 29
6.6 STUDY INTERVENTION COMPLIANCE 29
6.7 DOSE MODIFICATION 29
6.8 CONTINUED ACCESS TO STUDY INTERVENTION AFTER THE END OF THE STUDY 29
6.9 TREATMENT OF OVERDOSE 29
6.10 CONCOMITANT MEDICATIONS 29
6.11 PROHIBITED AND RESTRICTED THERAPIES DURING THE TRIAL 29
6.12 PERMITTED CONCOMITANT MEDICATIONS 30
7 DISCONTINUATION OF STUDY TREATMENT AND PATIENT DISCONTINUATION/WITHDRAWAL 30
8 STUDY ASSESSMENT AND PROCEDURES 30
8.1 SCREENING PROCEDURES 30
8.2 TRIAL PROCEDURES 31
8.3 SAMPLE REQUIREMENTS 36
8.4 DEVIATIONS AND SERIOUS BREACHES 38
9 SAFETY 39
9.1 SAFETY EVENT REPORTING: 39
9.2 REPORTING WINDOWS 39
9.3 ADVERSE EVENT TERM AND SEVERITY GRADE 39
9.4 SERIOUSNESS 39
9.5 CAUSALITY 39
9.6 EXPECTEDNESS ASSESSMENT: 40
9.7 REPORTING PROCEDURES 40
9.8 CLINICAL RESEARCH ORGANISTION RESPONSIBILITIES FOR SAFETY REPORTING TO REC 40
9.9 CLINICAL RESEARCH ORGANISTION RESPONSIBILITIES FOR SAFETY REPORTING TO MHRA 40
9.10 EMERGENCY UNBLINDING (PHASE II ONLY) 41

10 STATISTICS AND DATA ANALYSES 41
10.1 METHOD OF RANDOMISATION 41
10.2 SAMPLE SIZE 41
10.3 STATISTICAL ANALYSIS PLAN (SAP) 42
11 REGULATORY 43
11.1 CLINICAL TRIAL AUTHORISATION 43
12 ETHICAL CONSIDERATIONS 43
13 SPONSOR 43
13.1 INDEMNITY 43
13.2 FUNDING 43
14 TRIAL OVERSIGHT GROUPS 44
15 DATA MANAGEMENT 44
15.1 AUDITS AND INSPECTIONS 44
16 DATA SHARING REQUESTS FOR RESULTS THAT ARE AVAILABLE IN THE PUBLIC DOMAIN 44
17 MONITORING 44
18 RECORD RETENTION AND ARCHIVING 44
19 PUBLICATION POLICY 44
20 REFERENCES 45
21 SUMMARY OF SIGNIFICANT CHANGES TO THE CANDIDATE SPECIFIC TRIAL PROTOCOL 47

LIST OF ABBREVIATIONS SPECIFIC TO THIS CANDIDATE SPECIFIC TRIAL (CST) PROTOCOL

AE	Adverse event
ARDS	Acute respiratory distress syndrome
AUC	Area under the curve
BID	Twice daily
BP	Blood Pressure
CCI	Candidate Chief Investigator
CI	Chief Investigator
Cmax	Maximum concentration
COVID-19	Coronavirus - 19
CRO	Contract Research Organisation
CST	Candidate Specific Treatment
CTCAE	Common terminology criteria for adverse events
CV	Cardiovascular
CYP	Cytochrome P enzyme
DMEC	Data monitoring and ethics committee
DNA	Deoxyribonucleic Acid
EC	Effective Concentration
ECG	Electrocardiogram
ECMO	Extracorporeal membrane oxygenation
eCRF	Electronic Case Report Form
FI	Formalin-inactivated
GCP	Good Clinical Practice
GCS	Glasgow coma scale
GFR	Glomerular filtration rate
GP	General Practice (GP)
hOAT	human organic anion transporter
hURAT	Human Urate transporter
HR	Heart rate
ICU	Intensive Care Unit
IMP	Investigational Medicinal Product
IV	Intravenous
IWRS	Interactive web response system
LFT	Liver function test
MHRA	Medicines Healthcare regulatory authority
MRSA	Methicillin-resistant Staphylococcus aureus
NEWS2	National Early Warning Score 2
NIV	Non-invasive ventilation
NOAEL	No-observed-adverse-effect-level
PBMC	Peripheral Blood mononuclear cell
PCR	Polymerase Chain Reaction
PK	Pharmacokinetic
qSOFA	Quick Sepsis-Related Organ Dysfunction Assessment
RR	Respiratory Rate
RTP	ribonucleoside triphosphate
SAE	Serious adverse event
SAP	Statistical analysis plan
SAR	Serious adverse reaction
SCTU	Southampton Clinical Trial Unit
SUSAR	Suspected unexpected serious adverse reaction
SOC	Standard of Care
SRC	Safety Review Committee
SpO_2	Oxygen Saturation
T-705	Favipiravir
U&E	Urea & Electrolytes
UK	United Kingdom
URL	Uniform resource locator
UVA	Ultraviolet A
WHO	World Health Organisation
WOCBP	Women of childbearing potential (WOCBP)

1 CANDIDATE SPECIFIC PROTOCOL SUMMARY

1.1 CANDIDATE SPECIFIC SYNOPSIS

**Short title:**	**AGILE: Seamless Phase I/II Platform for the Rapid Evaluation of Candidates for COVID-19 treatment** - CST-6 Favipiravir
**Full title:**	**CST-6:** A Randomized, Multicentre, Seamless, Adaptive, Phase I/II Platform Study to Determine the Phase II dose and to Evaluate the Safety and Efficacy of intravenous (IV) Favipiravir for the Treatment of COVID-19

**Phase:**	Seamless Phase I/II
**Population:**	Adult in-patients (≥18 years) with laboratory confirmed COVID-19 infection by positive polymerase chain reaction (PCR) test. We will include patients defined according to the WHO Clinical Progression Scale (WHO, 2020) as follows:<br><br>**Group A and B (moderate to severe disease) – N.B. only Grades 4, 5 & 6 as defined by master protocol**<br>• Patients with clinical status of Grade 4 (hospitalised, no oxygen therapy), Grade 5 (hospitalised, oxygen by mask or nasal prongs) and Grade 6 (hospitalised, non-invasive ventilation or high flow oxygen)
**Primary Objective:**	**Phase I:**<br>• To determine the safety and tolerability of multiple doses of IV Favipiravir in patients with COVID-19<br>• To determine the maximum safe dose of IV Favipiravir for efficacy evaluation in phase II<br><br>**Phase II**:<br>• To investigate the efficacy of IV Favipiravir compared with placebo to reduce the SARS-CoV-2 viral load
**Secondary Objectives:**	**Phase I:**<br>• To characterise the plasma pharmacokinetics (PK) of multiple doses of IV Favipiravir<br>• To investigate the effect of IV Favipiravir on SARS-CoV-2 viral load<br>• To investigate the ability of IV Favipiravir to reduce the duration of signs and symptoms of COVID-19 in-patients.<br><br>**Phase II:**<br>• To determine the safety and tolerability of IV Favipiravir<br>• To characterise the plasma PK of IV Favipiravir<br>• To compare the effect of IV Favipiravir versus placebo on overall mortality, time to discharge, duration of oxygen use (and oxygen free days) and incidence and duration of new mechanical ventilation use<br>• To evaluate time to, and proportion of, clinical improvement (WHO clinical progression scale (WHO, 2020)).
**Exploratory Objectives:**	**Phase I & II:**<br>• To characterise the non-plasma PK of Favipiravir (saliva, tears, nasal secretions) and its active intracellular triphosphate in PBMC

	• To investigate the exposure-response relationship of IV Favipiravir on SARS-Cov-2 viral dynamics<br>• To characterise virus and host immune response<br>• Measure immune response by aldehyde oxidase (AO) / xanthine oxidase (XO) activity
**Rationale:**	Favipiravir is an antiviral agent with RNA-dependent RNA-polymerase (RdRp) inhibitory activity created by Fujifilm Toyama Chemical Co., Ltd. Favipiravir has been developed as an oral agent (**Oral Favipiravir**) licensed for influenza in Japan and is being evaluated in several COVID-19 clinical trials (Hassanipour, 2021). Favipiravir injection (**IV Favipiravir**) is a novel formulation of Favipiravir for intravenous drip infusion, with Cmax levels 4-fold higher following administration of multiple doses in cynomolgus monkeys compared to oral. The favipiravir activity is derived from the intracellular RTP metabolite that has a longer half-life intracellularly than the parent drug in plasma. Therefore, transiently higher Cmax values are expected to translate into sustained higher intracellular RTP concentrations and thus activity (Pertinez H., 2021).
**Trial Design:**	The first phase will be a 2:1 randomised open-label standard of care (SoC) controlled phase I of IV Favipiravir, followed by a 1:1 blinded, parallel group phase II trial of IV Favipiravir versus placebo (plus SoC). A phase I will be carried out to test the safety and tolerability of IV Favipiravir in hospitalised patients.<br><br>Following review of safety, tolerability and PK data from evaluated phase I doses, an IV Favipiravir dose will be selected to progress to phase II. The selected dose of IV Favipiravir will be evaluated in a blinded, placebo controlled randomised phase II trial, which will assess the safety and virological efficacy.
**Sample size:**	**Phase I:**<br>The plan is to test up to 5 dose levels of IV Favipiravir in up to 5 cohorts of 6 patients each (30 patients in total). Fewer than 5 cohorts and up to 42 patients (allowing for another two cohorts of 6 participants) may be enrolled, with the final number dependent on dose escalation decisions. Each cohort includes 4 IV Favipiravir patients and 2 SoC.<br><br>**Phase II:**<br>A maximum of 198 participants, randomised in a 1:1 ratio IV Favipiravir and placebo, will be enrolled in the phase II stage. There will be a blinded efficacy and futility review after the first 66 patients (one third of the way through the trial without pausing the recruitment).
**Investigational Medicinal Product/s:**	Favipiravir: 6-fluoro-3-hydroxypyrazine-2-carboxamide, T-705<br><br>Phase I: IV Favipiravir or SoC (open label)<br>Phase II: IV Favipiravir or placebo (double blinded)<br><br>Favipiravir is a novel nucleic acid (pyrazine molecule) analogue that interferes with viral ribonucleic acid (RNA) replication and has demonstrated activity against SARS-CoV-2 both in-vitro and in animal infection models (Wang, 2020) (Suzanne, 2020).
**Dosage Regimen / Duration of Treatment:**	**Phase I:**<br>Multiple doses of IV Favipiravir will be administered by intravenous (IV) infusion over 1 hour. Dosing regimen will be every 12 hours for 7 days duration. The starting dose will be 600mg (BID), and dose escalations to 1200mg (BID), 1800mg (BID) and 2400mg (BID) are anticipated as well as a de-escalation dose of 300mg (BID) if necessary, with de-escalation and

	escalation guided by emerging safety data and decision by the Safety Review Committee (SRC). Duration of monitoring will be 29 days post-first dose.<br><br>**Phase II:**<br>An IV Favipiravir dose will be selected depending on safety, tolerability and PK data from phase I. The IV Favipiravir dose will be given by IV infusion BID over 1 hour with matching placebo.

**URL for Databases and Randomisation:**	Sctu.mdsol.com

**Primary Trial Endpoints:**	**Phase I:**<br>• Adverse events and serious adverse events<br>• Dose limiting toxicities (Safety and Tolerability of IV Favipiravir – CTCAE v5 Grade ≥3 adverse events possibly or probably related to the IMP) up to day 8<br><br>**Phase II:**<br>• Change from baseline to Day 3 in SARS-CoV-2 viral load
**Secondary Trial Endpoints:**	**Phase I:**<br>• Plasma PK parameters of Favipiravir up to Day 8<br>• Change from baseline over time, up to Day 29, in viral load<br>• WHO Clinical Progression Scale at day 15 and 29<br>• Mortality at Days 15 and 29<br>• Time from randomisation to death (up to day 29)<br><br>**Phase II:**<br>• AEs, SAEs, physical findings, vital signs and laboratory parameters<br>• Plasma PK parameters of IV Favipiravir up to Day 8<br>• Change from baseline over time, up to Day 29, in viral load<br>• Time to negative viral titres (Day 1-29)<br><br>• Time to and proportion of clinical improvement. Improvement will be determined according to the WHO Clinical Progression Scale (WHO, 2020); improvement is defined as a minimum 2-step change from randomisation in the scale up to day 29 post-randomisation:<br>• Proportion of patients with clinical improvement (as defined above) at day 8, 15 and day 29<br>• Change at day 8 and 15 from randomisation in the WHO Clinical Progression Scale (WHO, 2020)<br>• Time to a one point change on the WHO Clinical Progression Scale (WHO, 2020)<br><br>• To evaluate the time to, and proportion of discharge:<br>• Time to discharge from randomisation<br>• Proportion of patient discharged by days 8, 15 and 29<br><br>• To evaluate overall mortality:<br>• Mortality at Days 8, 15 and 29<br>• Time to death from randomisation<br>• Duration (days) of oxygen use and oxygen free days<br>• Incidence of new mechanical ventilation use and duration (days) of new mechanical ventilation use

	• Time to death from randomisation
**Exploratory endpoints:**	**Phase I & II:**<br>• Concentrations of IV Favipiravir in saliva, tears, and nasal mucosal secretions & Dried Blood Spots (DBS)<br>• Concentrations of Favipiravir ribonucleoside triphosphate (FAVI-RTP) in cells<br>• Change in host immune response and SARS-CoV-2 culture and sequencing<br>• To investigate the exposure-response relationship of IV Favipiravir on SARS-Cov 2 viral dynamics
**Total Number of Sites:**	3-8 UK Clinical Research Facilities

1.2 CANDIDATE SPECIFIC TRIAL SCHEMA

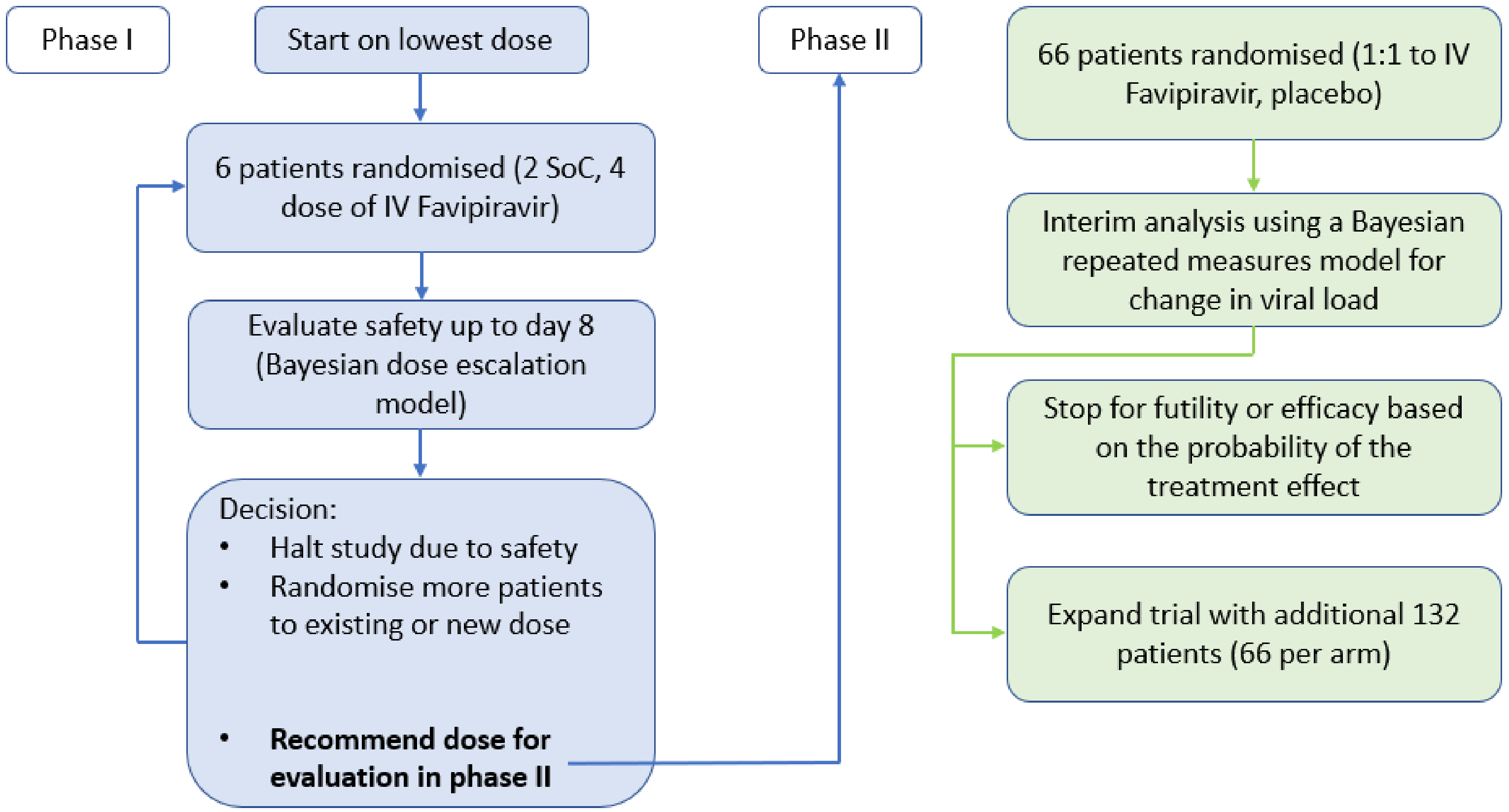

1.3 SCHEDULE OF OBSERVATIONS AND PROCEDURES (Phase I and II)

	Screening[k]	Day 1 (baseline)	Daily whilst in hospital (days 2-14)	Specific Assessments on Days 3, 5, 8, 11 (±1 days)	Day 15 (±2 days)	Day 29 (±2 days)
**Informed consent**	X					
**Check Eligibility**	X[a]	X				
**Randomisation**		X				
**Telephone Contact[b] (AEs, Con-Meds)**			Daily apart from in hospital			
**Demographics**	X					
**Treatment compliance - up to day 7**			X (Day 2 to 7)			
**AE/SAE assessment**	X (from consent)	X	X	X	X	X
**12 lead ECG[i]**	X					
**SARS-CoV-2 surveillance NP swab**		X	Daily for 1st 5 days	X	X	X
**SARS-CoV-2 translational NP swab**		X		X[c]		
**Full Blood Count**	X[d]	X	As per SoC	X	X	X
**U&Es**	X[d]	X	As per SoC	X	X	X
**Uric acid**	X[d]	X	As per SoC	X	X	X
**Estimated GFR**	X[d]	X	As per SoC	X	X	X
**LFTs**	X[d]	X	As per SoC	X	X	X
**PK assessment (Plasma, dried blood spots)**		X[e]		X[e]		
**PK assessment (tears, saliva, nasal secretions, PBMC)**		X[fg]		X[fg]		
**Pregnancy test WOCBP**	X (serum)					X (urine)
**Urinary analysis**	X[d]	X				X
**Medical history (including COVID-19 history)**	X					
**Con-med/SoC review**	X	X	X	X	X	X

	Screening[k]	Day 1 (baseline)	Daily whilst in hospital (days 2-14)	Specific Assessments on Days 3, 5, 8, 11 (±1 days)	Day 15 (±2 days)	Day 29 (±2 days)
**Height/Supine length/Weight** [l]	X					
**WHO Progression scale**	X	X		X	X	X
**Vital signs[j] (NEWS2, HR, BP, RR, sats & temp)**	X	X	X	X	X	X
**Assessment of oxygen use (low flow, high flow, mechanical ventilation)**	X[h]	X[h]	X[h]		X (if hospitalised)	
**Targeted physical exam**	X	X	X	X	X	X
**Chest X-ray/other chest imaging**	If clinically indicated					
**Blood collection for translational research**		X (pre dose)		X	X	X
**End of study**						X

a. Can be conducted on Day 1 but must be prior to randomisation to confirm eligibility
b. Additional week 24 telephone consultation for pregnancy follow up if female participants became pregnant.
c. Day 3 and 5 only.
d. Results are required prior to dosing only if clinically indicated
e. PK sampling for Phase I: Day 1 (pre-dose, 0-1 hr, 2-4 hr and 6-12 hr post completion of first infusion), Day 3 (pre-dose, 0-1 hr, 2-4 hr and 6 -12 hr post completion of first infusion) and at Day 5 (0-1 hr and 6-12 hr post completion of the first infusion). **NOTE:** 6-12 hours post infusion must be taken prior to second infusion
f. Non-plasma PK sampling for Phase I: tears, saliva, nasal collections Day 1 and Day 3 (6-12 hours post completion of infusion)
g. Intracellular (PBMC) sampling for Phase I: Day 1 and Day 3 (6-12 hr post completion of first infusion) and at Day 5 (6-12 hr post completion of the first infusion)
h. If supplemental oxygen not being administered, lowest oxygen saturation over the last 24 hours to be recorded as part of baseline/screening
i. 12-lead ECG and 10 second ECG rhythm strip (≥5 min supine), to be performed during screening, or on baseline day 1 prior to first dose
j. Vital signs on Day 1 are collected prior to dose administration (as per AGILE CST6 Acceptable Time Windows Working Instruction)
k. Screening and Day 1 can occur on the same day, therefore if not already done at screening, perform all screening procedures at baseline if on the same day, if not, then repeat on baseline as per SoA.
[l] Height and weight may be taken from current admission

2 INTRODUCTION

2.1 BACKGROUND AND RATIONALE

A novel betacoronavirus was first reported in December 2019 causing severe pneumonia in Wuhan, China. Since that time, Severe Acute Respiratory Syndrome coronavirus-2 (SARS-CoV-2) has spread throughout the world leading to over 600 million confirmed cases and over 3 million deaths as of September 2022 (Hopkins, 2021). Mortality estimates from the early in the pandemic There is an urgent need for effective treatment throughout the course of illness. Current literature suggested a case fatality rate of exceeding that of seasonal influenza by over 10-fold, ranging from 1.8 to 3.4% overall (Ferguson, 2020) (Bialek, 2020). Morbidity and mortality increased markedly with advanced age and comorbidities to 25% in some patient populations (Ferguson, 2020) (Bialek, 2020). Although only approximately 25% of infected patients have comorbidities, 60-90% of hospitalized infected patients had comorbidities, the most common of which include hypertension, obesity, chronic lung disease, diabetes and cardiovascular disease. Additionally, hospitalization rates appeared to be highest among those over age 65 (Wiersinga, 2020). Among hospitalized patients, between 17% to 35% of those who were hospitalized will require critical care and that 50% of those in critical care would die (Ferguson, 2020) (Wiersinga, 2020). In addition, an age-dependent proportion of those that did not require critical care would also die (Ferguson, 2020).

As we approach 3 years since the initial emergence of SARS-CoV-2, a number of factors have had a substantial impact on case fatality rates, hospitalisations and deaths due to Coronavirus disease 2019 (COVID-19) (ref). These include increased population immunity through both mass vaccination and natural exposure to SARS-CoV-2, emergence and predominance of variants with lower virulence, and the development of effective therapeutics for treatment of COVID-19. However, the need for development of effective anti-viral therapeutics remains.

Dexamethasone and IL-6 inhibitors such as Tocilzumab have been shown by large phase III platform clinical trials to improve outcomes in hospitalised patients with severe COVID-19 (requiring supplemental oxygen) and have become standard of care in many countries as recommended by WHO. A number of antiviral therapeutics are also recommended for use by WHO in non-severe COVID-19 including Paxlovid (Nirmatrelvir/ritonavir), Molnupiravir and Remdesivir (WHO, 2022). Many monoclonal antibody therapeutics, evaluated in large platform trials and given approval for use, have in many settings now been withdrawn due to a lack of efficacy against new variants.

There remains an important medical need for anti-viral therapeutics for the treatment of COVID-19. Antiviral treatment of severe disease in hospitalised patients may prevent progression to more severe sequelae of COVID-19 including respiratory failure, and non-respiratory complications of COVID-19 including thromboembolic disease leading to pulmonary embolism and stroke, arrhythmias, and shock, among others (Klok, 2020) (Chen, 2020). It may also reduce length of stay in overwhelmed treatment facilities and furthermore, antiviral treatment could potentially reduce risk of nosocomial transmission. Early anti-viral treatment of non-severe COVID-19 may reduce the risk of progression to severe disease and hospitalisation in patients at high risk either due to co-morbidity or immunocompromise (Gottlieb, 2022).

Favipiravir (6-fluoro-3-hydroxypyrazine-2-carboxamide; T-705) has been shown to be an antiviral drug with broad spectrum capabilities in non-clinical studies and has been investigated in clinical studies for its efficacy in uncomplicated influenza in adult patients. Favipiravir has also been employed in the treatment of Ebola in a clinical trial sponsored by the French Government in Guinea during the 2014

epidemic, where favipiravir showed suggestions of efficacy. Additional indications under consideration or used under compassionate use include severe fever with thrombocytopenia virus, rabies, Lassa fever, Jamestown Canyon virus and norovirus (Furuta, 2017).

FAVIPIRAVIR AND COVID-19

Phase I of this AGILE study will comprise a dose-escalation phase for the IV formulation of Favipiravir, in which patients will be randomized to IV Favipiravir or SoC in a 2:1 allocation ratio. Each of these cohort's safety data will be reviewed between cohorts by the Safety Review Committee (SRC). Based on review of safety and tolerability data from evaluated doses, a dose of IV Favipiravir (up to 2400 mg BID) will progress to Phase II, which will evaluate IV Favipiravir and placebo in a 1:1 ratio. The primary aim of this study is to assess the safety, tolerability and virological efficacy of IV Favipiravir compared with placebo.

Significant uncertainty exists about the suitability and efficacy of Favipiravir as a COVID-19 therapeutic intervention. Teratogenicity concerns are well established and the high pill burden may limit widespread uptake of the drug during early infection, particularly in the absence of concomitant contraceptive use in women of child-bearing age. Furthermore, in vitro studies of Favipiravir in Vero-E6 cell infected with SARS-CoV-2 have yielded inconsistent findings (Choi, 2020) (Hattori, 2020) (Choy, 2020) (Wang, 2020), and low potency (EC90 = 159 µM; 24.9 µg/mL) has been described in those studies that have shown activity.

Favipiravir has demonstrated robust antiviral activity in the Syrian hamster model of SARS-CoV-2 infection, having a marked effect upon viral RNA, infectious virus titres and pulmonary disease pathology (Kaptein, 2020) (Driouich, 2021). A number of clinical trials of oral Favipiravir in patients with COVID-19 have been undertaken with mixed results. A phase III randomised placebo-controlled clinical trial of oral Favipiravir (1800 mg BID on Day 1 followed by 800 mg BID up to Day 14) in mild/moderate disease severity hospitalized COVID-19 patients in Japan demonstrated improved time to clinical improvement and SARS-CoV-2 viral clearance (FUFIFILM Toyama Chemical Co., 2020). In India a phase III trial of 150 patients with mild/moderate disease showed improved time to clinical improvement with Favipiravir (Udwadia, 2020), a small trial in hospitalised patients in Russia demonstrated an improved proportion of patient with viral clearance on Favipriavir compared to SoC (Ivashchenko, 2020) and an RCT in China showed no difference in clinical recovery compared to arbidol (Chen, 2020). It should be noted that the doses used in the Chinese trial (1600mg BID day 1 followed by 600mg BID maintenance) are lower than those studied in the other trials (1800mg BID day 1 followed by 800mg BID maintenance).

2.2 RISK/BENEFITS FOR TREATMENT ARM / IMP

Pharmacology and pharmacokinetics

US Adopted Name/ International non-proprietary name:	Favipiravir
Chemical name:	6-fluoro-3-hydroxypyrazine-2-carboxamide
Lab Code:	T-705
Chemical Abstracts Service number:	259793-96-9
Molecular formula:	C5H4FN3O2
Molecular weight:	157.10
Description:	Favipiravir is a white to light yellow powder. Favipiravir is sparingly soluble in acetonitrile and methanol, and slightly soluble in ethanol and water.
Melting point:	191°C

Stability: Favipiravir is stable for 5 years at 15 to 30° C (59 to 86° F).

Chemical structure of Favipiravir

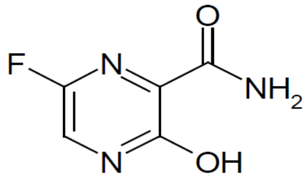

In adults, oral Favipiravir is rapidly and completely absorbed following administration. Across the dose range and formulations utilized, the time to maximum plasma concentration (Tmax) of Favipiravir occurs between 0.5 to 4.0 hours, the median time being 1.0 hour after a single dose of Favipiravir and 2.0 hours following multiple doses. A repeat-dose study over 5 days has shown that Favipiravir in plasma accumulates due to an inhibition of the major drug metabolizing enzyme, aldehyde oxidase, by Favipiravir.

Favipiravir administered twice daily (BID) resulted in a greater than proportional increase in Favipiravir blood levels. The average dose-adjusted Day 5 area under the plasma concentration vs time curve (AUC) ratio comparing 600 mg BID with 800 mg BID was 1.5 in one study and 1.9 in a second study of healthy volunteers. Favipiravir plasma protein binding averages 53 to 54%, of which 65% is bound to albumin and 6.5% to alpha-1-acid glycoprotein. Favipiravir, metabolite (hydroxide) of T-705 (T-705M1) and glucuronide conjugate of T-705 (T-705M2) are excreted predominately in urine and to a small extent into the feces. In healthy volunteers following administration of a single oral 400 mg dose, 86.7% of the dose was recovered in the urine as the T-705M1 metabolite, 3.6% as a glucuronide conjugate, and 0.2% as Favipiravir, totaling 90.5% at 48 hours.

The major metabolite, T705M1, is formed by aldehyde oxidase in human liver cytosol and other tissues. The cytochrome mixed function oxidase systems do not significantly contribute to the metabolism of Favipiravir. Favipiravir exhibits only weak inhibitory effects on cytochrome P450 (CYP) 1A2, 2C9, 2C19, 2D6, 2E1, or 3A4 (inhibitory concentration to 50% [IC50] >800 µmol/L, 126 µg/mL). Favipiravir does inhibit CYP2C8 in human liver microsomes. Favipiravir showed little or no induction of CYP1A2, CYP2C9, CYP2C19 and CYP3A4 in human hepatocytes. Both Favipiravir and T-705M1 moderately inhibit human organic anion transporter (hOAT) 1, hOAT3, and human urate transporter (hURAT) 1 (30.9 to 65.7% of control). Additionally, hURAT1-mediated uric acid uptake was increased by T-705M1, suggesting T-705M1 may stimulate the reabsorption of uric acid in human renal proximal tubules. Food interaction studies have shown Favipiravir absorption from the 200 mg tablet formulation was not altered when taken with a high fat meal. The extent of absorption of Favipiravir plasma concentrations over time, as determined by AUC, was 316.3 µg hr/mL vs. 299.8 µg hr/mL following tablet administration alone and with food, respectively.

No alteration of dosing is needed in patients with renal impairment. Total (AUCinf) exposure for plasma Favipiravir for patients with severe renal impairment (Stage 4) was 1.3-fold higher compared to patients with normal renal function. No obvious effect of renal impairment on safety was observed and

Favipiravir treatment was generally well tolerated in patients with renal impairment. No data on renal failure with or without dialysis are available. The maximum plasma concentration (Cmax) and AUC values in elderly patients in a single and a multiple-dose study completed in Japan were higher than in young patients. Comparing AUC values on Day 5, the differences were 40 and 80% after 600 mg once a day and 400 mg BID, respectively. In the companion study completed in the United States of America (US), there were no differences between young and elderly populations based on Day 5 AUC comparison in patients receiving either 600 or 800 mg BID. Previous reports have suggested that Favipiravir PK exposures demonstrated may be lower in American and African patients compared to Chinese patients (Nguyen TH, 2017) . More recent modelling suggests no significant ethnic differences.

Toxicology

Non-clinical studies

Repeat dose toxicity studies consist of 28-day studies in rats and dogs and 14 days in monkeys. For rats, the NOAEL was 32 mg/kg/day. The threshold effect level was 80 mg/kg/day, at which reduction in weight gain and minor alterations in various clinical pathology parameters were observed. Mortality was seen at 200 mg/kg/day. Histopathological effects were limited to decreased hematopoiesis.

In the 28-day dog study, the NOAEL was 10 mg/kg/day. The major effect was in the male reproductive organs where decreased weights were seen in the testes and accessory glands at all doses, particularly at 100 mg/kg/day. These findings were not statistically significant nor dose related. Histopathological effects (hypospermatogenesis) were limited to the high dose of 300/100 mg/kg/day. The other threshold effect level was 30 mg/kg/day where emesis and decreased weight gain were noted. Mortality was seen at 300 mg/kg/day. In monkeys, the NOAEL was 100 mg/kg/day following 14 days of treatment. At higher doses, body weight gain was reduced and some alterations in clinical pathology parameters were noted. Histopathological effects were limited to vacuolar degeneration of hepatocytes and minor necrotic changes in the seminal vesicles in some but not all males. The testicular effects seen in the dog were not observed in the monkey.

Embryo-fetal development was evaluated in mouse, rat, rabbit, and monkey. Teratogenicity occurred in all species at doses causing mild to marked maternal toxicity and was accompanied by embryolethality in rat and mouse but not in rabbit or monkey. Fetal malformations occurred at doses of 300, 100, 600, and 200 mg/kg/day in the mouse, rat, rabbit, and monkey, respectively. NOAELs for developmental toxicity, based primarily on fetal body weight changes, were 100, 20, 300, and 100 mg/kg/day in those species, respectively, and were similar to NOAELs demonstrated for general toxicity in 14- or 28-day pivotal studies. When compared to two other antiviral drugs, Favipiravir had a margin of safety (ie, low multiples of clinical exposure) for developmental toxicity similar to valacyclovir (Pregnancy Category B) and a more favorable margin than ribavirin (Pregnancy Category X), which caused teratogenicity at subclinical exposures. Early embryonic and embryo-fetal developmental toxicity studies with the T-705M1 metabolite revealed no teratogenicity or other developmental effects at doses of up to 100 mg/kg/day in rats and 300 mg/kg/day in rabbits. Based on dose range finding studies in rabbits and cesarean section data from the definitive study in rabbits, T-705M1 does not appear to be teratogenic in rabbits. The T-705M1 teratology studies in rabbits provided the rationale for contraception period reduction from three months to seven days after the last dose for both men and women.

Phototoxic effects have been seen with Favipiravir in mice at 100 mg/kg and 300 mg/kg groups and in guinea pigs at 100 mg/kg. In mice at the 100 mg/kg dose, erythema was observed at 0.5 and 24 hours after ultraviolet A (UVA) irradiation and was alleviated or disappeared at 48 and 72 hours. In the 300 mg/kg dose in mice, erythema was observed at 0.5 to 72 hours after UVA irradiation. No skin reactions were observed in non-irradiated animals receiving favipiravir. In guinea pigs, slight erythema was noted at 24 hours after UVA exposure but subsided after 48 hours. Genotoxicity studies have been conducted

with Favipiravir, and additional studies were conducted evaluating T-705M1. Three studies were negative: the Ames test; the in vivo micronucleus assay in rats, following two daily doses at up to 1000 mg/kg (a near lethal dose of 2000 mg/kg produced a positive response by a secondary mechanism); and the in vivo unscheduled DNA synthesis assay, conducted in rats at single doses up to 2000 mg/kg.

Clinical studies

In total 33 phase I studies have been conducted in the US or Japan during which the safety profile appeared similar to that of placebo and comparator agents with the exception of asymptomatic elevations in uric acid levels. Elevations in blood uric acid trended towards baseline or within normal ranges after Favipiravir study dosing completed and, therefore, were not considered clinically significant. No other clinically notable shifts in serum chemistry, hematology/coagulation, urinalysis, or other special laboratory parameters were observed. To date, Favipiravir has not been shown to have any clinically significant effect on QT prolongation, or in special populations such as the elderly or patients with renal impairment. Dosage adjustment of Favipiravir should be considered for patients with hepatic impairment.

Three phase II studies in patients with uncomplicated influenza have been completed with favipiravir's safety profile similar to that of placebo with the exception of asymptomatic elevations in uric acid. Four phase III studies in patients with uncomplicated influenza have been completed. As was noted in earlier phase studies, Favipiravir administration was associated with asymptomatic elevations in uric acid. Uric acid elevations were noted at Day 5 and resolved or trended towards baseline by the first post-dosing assessment time point on Day 15. At baseline, the mean uric acid level for the Favipiravir group was 0.263 mmol/L for females and 0.342 mmol/L for males. At Day 5, the mean uric acid level for the Favipiravir group was 0.417 mmol/L for females and 0.448 mmol/L for males. At Day 15 (the first post-treatment time point analyzed), the mean uric acid level had fallen to 0.288 mmol/L for females and 0.368 mmol/L for males. The elevations in uric acid were not associated with any clinically identifiable AE known to be associated with acute or chronic elevations of uric acid (e.g. gout, urate nephropathy). Such AEs are known to develop after notably higher uric acid elevations over considerably longer periods of time.

In a two-part, blinded study (JP115), the effects of Favipiravir on the QT/QTc interval in healthy volunteers were evaluated. Part A (n=12) of the study assessed the tolerability, safety, and PK of a single 2000 mg or 2400 mg dose of Favipiravir. Part B (n=56) assessed the QT/QTc intervals of 1200 mg Favipiravir, 2400 mg Favipiravir, placebo, or moxifloxacin 400 mg as the positive control in a cross-over design with at least a 14-day washout period. Plasma Favipiravir concentration reached peak value two to three hours after administration. The maximum estimated values for ΔQTc (Fridericia) with Favipiravir 1200 and 2400 mg were 0.83 msec (3 hours after administration) and 0.50 msec (6 hours after administration), respectively. The maximum values of the upper limit of the one-sided 95% CI with Favipiravir 1200 and 2400 mg were 3.17 msec (6 hours after administration) and 2.88 msec (6 hours after administration), respectively. At any time point, the upper limit of the one-sided 95% CI of ΔΔQTc (Fridericia) estimate was less than 4 msec, which satisfied previously defined criteria that indicates no prolongation effect on QT/QTc interval (upper limit of the one-sided 95% CI of ΔΔQTc is below 10 msec). A single dose of Favipiravir at 1200 or 2400 mg had no prolongation effect on QT/QTc interval.

Testicular safety studies in healthy males to evaluate the effects of Favipiravir versus placebo on a regimen of 1200 mg twice daily (BID) for 1 day followed by 800 mg BID for 4 days found no statistically significant differences between favipiravir and placebo for changes from baseline semen parameters, and semen favipiravir concentrations were below the limit of quantitation by 7 days after last dose.

A Japanese investigator-conducted program in patients with severe fever with thrombocytopenia syndrome virus administered 1800 mg BID Day 1 followed by 800 mg BID Days 2-10. Patients appeared to tolerate this well. Inserm has conducted an open label clinical trial in Ebola patients in West Africa (the JIKI trial (Nguyen TH, 2017)) and the doses of 2400 mg, 2400 mg and 1200 mg on Day 1 (given every eight hours) followed by 1200 mg BID for up to an additional nine days, appeared to be well tolerated (Sissoko, 2016). A small number of patients with a variety of RNA viral diseases (rabies, Lassa fever, norovirus) have been treated at different dose regimens under compassionate use. None of these regimens exceeded the doses given in the JIKI trial, nor exceeded the 21 days given in JP120.

PKPD modelling

A key principle for antiviral drugs is that the plasma drug concentrations are maintained above the in-vitro defined target concentrations for the duration of the dosing interval. Modelling based on published plasma PK profiles and the likely concentrations of the intracellular active form (FAVI-RTP) have demonstrated necessary doses required to achieve target concentrations to inhibit SARS-CoV-2.

Simulations have indicated that favipiravir maintenance doses between 800mg and 1200mg BID may be sufficient to provide therapeutic concentration of FAVI-RTP during the dosing period.

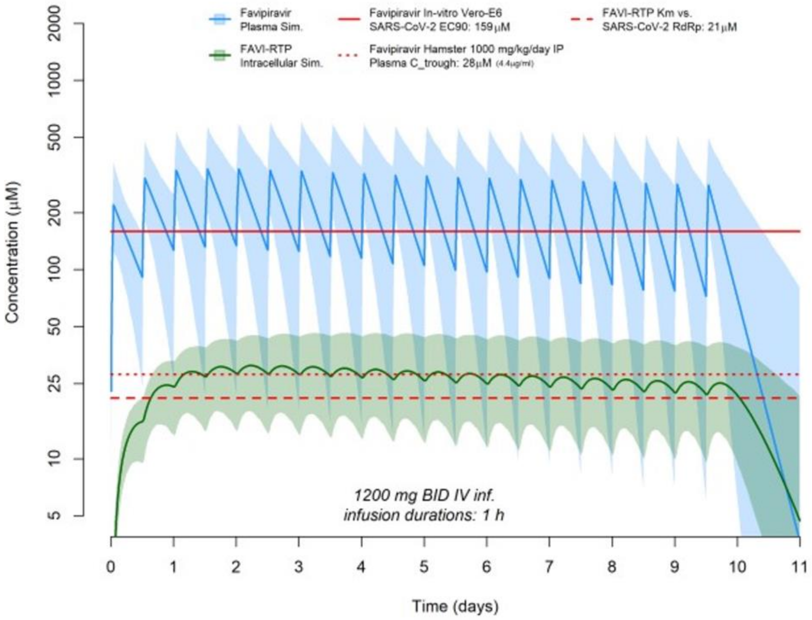

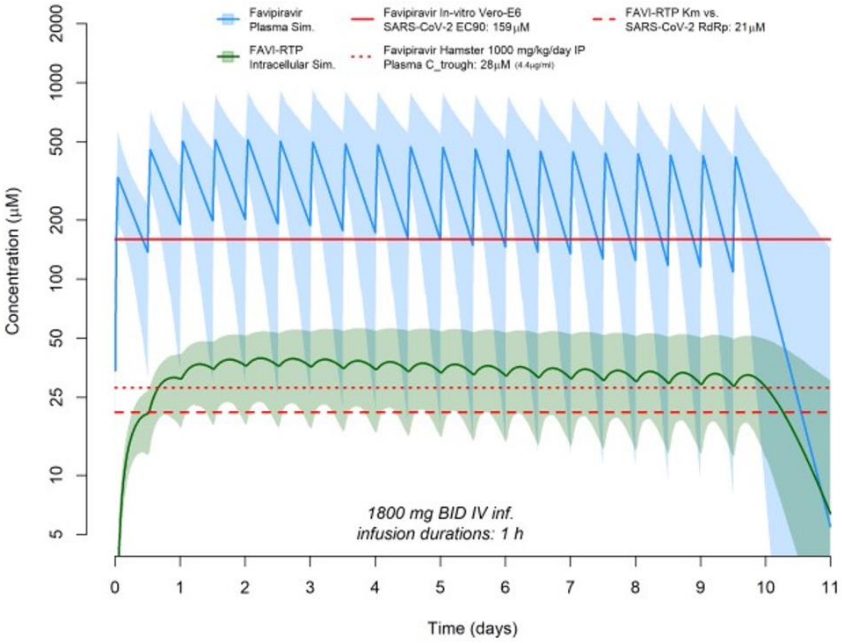

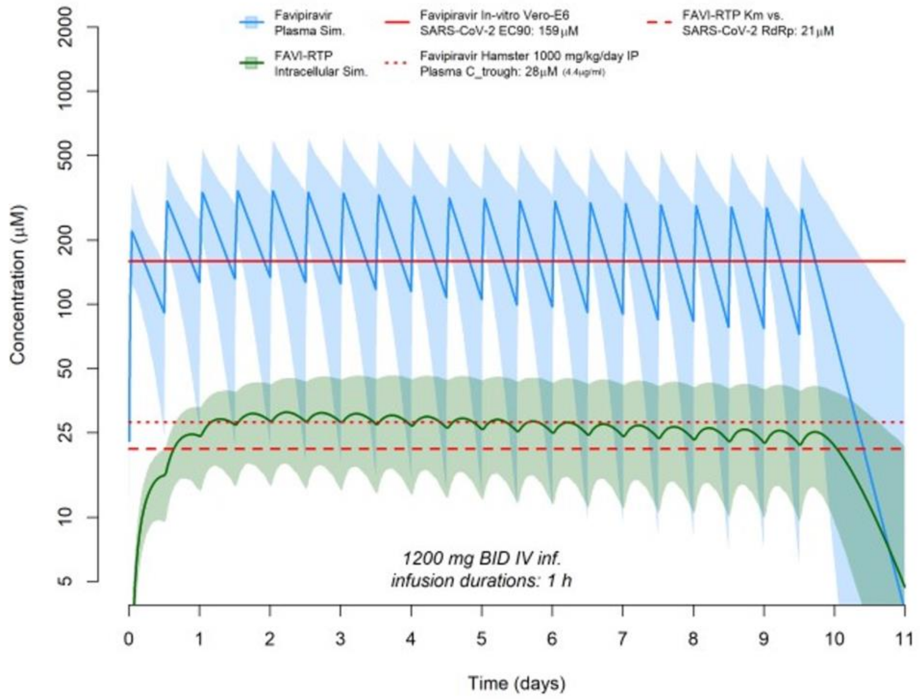

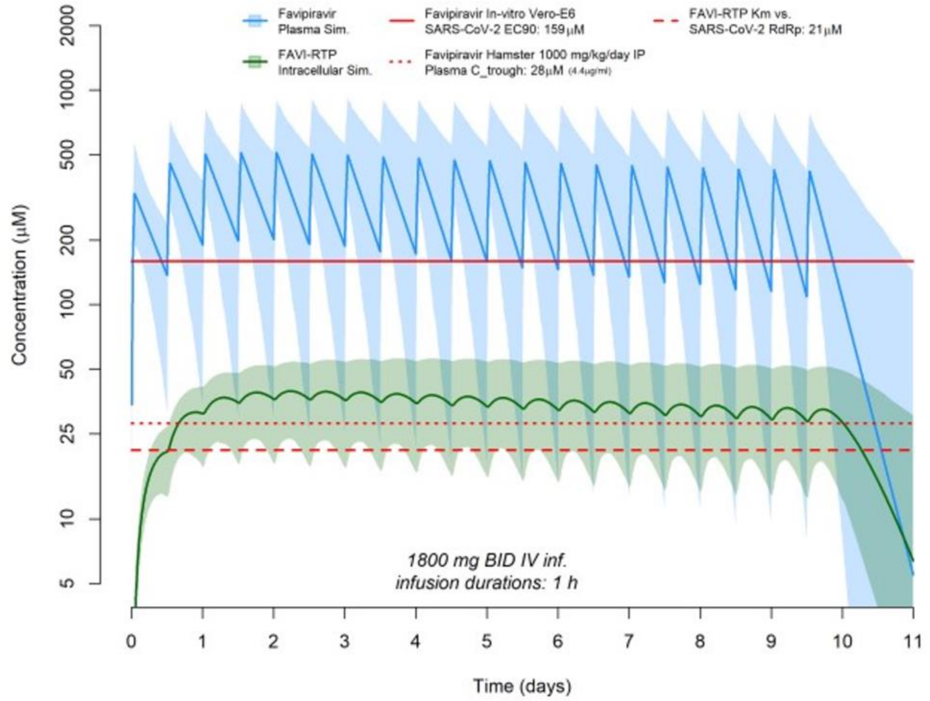

Figure. Favipiravir IV Infusion human PK simulations (CLIV = 2.66 L/h, V = 33.39 L)
Validated using a previously published model with input data from Wang et al (Wang, 2020). PO apparent CL/F and V/F, with F assumed = 0.9
Intracellular prediction from in-vitro extrapolation

Overall Benefit: Risk Conclusion

Based on the high unmet medical need and considering the measures taken to minimize risk to participants participating in this study, the potential risks identified in association with Favipiravir are justified by the anticipated benefits that may be afforded to participants with moderate to severe COVID-19.

3 CANDIDATE SPECIFIC OBJECTIVES AND ENDPOINTS

Note that this CST has additional objectives to the AGILE Master Protocol, and includes only those endpoints related to the Group A patient (Grades 5 and 6) and hospitalised group B patient (Grade 4) population being evaluated in this CST.

3.1 PHASE I OBJECTIVES & ENDPOINTS

Objectives	Endpoints
**Primary**	
**Safety objective:** To determine the safety and tolerability of multiple doses of IV Favipiravir in patients with COVID-19<br><br>**Efficacy objective**: To determine the maximum safe dose of IV Favipiravir for efficacy evaluation in phase II	• Adverse events and serious adverse events<br>• Dose limiting toxicities (Safety and Tolerability of IV Favipiravir– CTCAE v5 Grade ≥3 adverse events possibly or probably related to the IMP) up to day 8
**Secondary**	
**Pharmacokinetic objective:** To characterise the pharmacokinetics (PK) of multiple doses of IV Favipiravir	• Plasma PK parameters of IV Favipiravir up to Day 8
**Virologic objective**: To investigate the effect of IV Favipiravir on SARS-CoV-2 viral load	• Change from baseline over time, up to Day 29, in viral load
**Clinical objective:** To investigate the ability of IV Favipiravir to reduce the duration of signs and symptoms of COVID-19 in-patients.	• WHO Progression Scale (WHO, 2020) at day 15 and 29:<br><br>0. Uninfected, no viral RNA detected<br>1. Ambulatory mild disease, asymptomatic; viral RNA detected<br>2. Ambulatory mild disease, symptomatic; independent<br>3. Ambulatory mild disease, symptomatic; assistance needed<br>4. Hospitalised moderate disease, no oxygen therapy (If hospitalised for isolation only, record status as for ambulatory patient)<br>5. Hospitalised moderate disease, oxygen by mask or nasal prongs<br>6. Hospitalised severe disease, oxygen by NIV or high flow<br>7. Hospitalised severe disease, intubation and mechanical ventilation, pO_2/FiO_2 ≥150 or SpO_2/FiO_2 ≥200<br>8. Hospitalised severe disease, mechanical

	ventilation pO_2/FiO_2 <150 (SpO_2/FiO_2 <200) or vasopressors<br>9. Hospitalised severe disease, mechanical ventilation pO_2/FiO_2 <150 and vasopressors, dialysis, or ECMO<br>10. Dead<br><br>• Mortality at Days 15 and 29<br>• Time from randomisation to death (up to day 29)
**Exploratory**	
**To characterise the non-plasma PK of Favipiravir (saliva, tears, nasal secretions) and its active intracellular triphosphate in PBMC**<br><br>To investigate the exposure-response relationship of IV Favipiravir on SARS-Cov-2 viral dynamics<br><br>To characterise virus and host immune response	• Concentrations of IV Favipiravir in saliva, tears, and nasal mucosal secretions & Dried Blood Spots (DBS)<br>• Concentrations of Favipiravir ribonucleoside triphosphate (FAVI-RTP) in cells<br><br>• Change in host immune response and SARS-CoV-2 culture and sequencing<br>• Measure immune response by aldehyde oxidase (AO) / xanthine oxidase (XO) activity

3.2 PHASE II OBJECTIVES & ENDPOINTS

Objectives	Endpoints
**Primary**	
To investigate the efficacy of IV Favipiravir compared with placebo to reduce the SARS-CoV-2 viral load	• Change from baseline to Day 3 in SARS-CoV-2 viral load
**Secondary**	
**Safety objective**: To determine the safety and tolerability of IV Favipiravir	• AEs, SAEs, targeted physical findings, vital signs and laboratory parameters
**Pharmacokinetic objective**: To characterise the plasma PK of IV Favipiravir	• PK parameters of IV Favipiravir up to Day 8
**Virological objective:** To investigate the efficacy of IV Favipiravir on SARS-CoV-2 viral load	• Change from baseline over time, up to Day 29, in viral load<br>• Time to negative viral titres (Day 1-29)
**Clinical objectives:**<br>To compare the effect of IV Favipiravir versus placebo on overall mortality, time to discharge, duration of oxygen use (and oxygen free days) and incidence and duration of new mechanical ventilation use<br><br>To evaluate time to, and proportion of, clinical improvement (WHO clinical progression scale).	• Mortality at Days 8, 15 and 29Time to death from randomisation)<br>• Proportion of patient discharged by days 8, 15 and 29 (Time to discharge from randomisation)<br>• Duration (days 1 - 7) of oxygen use and oxygen-free days.<br>• Duration (days) of mechanical ventilation<br>• Incidence of new mechanical ventilation use and duration (days) of new mechanical ventilation use.<br>• Change from baseline over time, up to Day 29, in viral load<br>• Time to negative viral titres (Day 1-29)<br>• Time to and proportion of clinical improvement. Improvement will be determined according to the WHO Clinical Progression Scale (WHO, 2020); improvement is defined as a minimum 2-

	step change from randomisation in the scale up to day 29 post-randomisation<br>- Proportion of patients with clinical improvement (as defined above) at day 8, 15 and day 29<br>- Change at day 8 and 15 from randomisation in the WHO Clinical Progression Scale (WHO, 2020)<br>- Time to a one point change on the WHO Clinical Progression Scale at day 15 and 29
**Translational**	
Pharmacodynamics: To characterise virus and host immune response	• Change in host immune response and SARS-CoV-2 culture and sequencing (samples will be stored pending the availability of GCP-compliant assays which are currently in development)<br>• Measure immune response by aldehyde oxidase (AO) / xanthine oxidase (XO) activity

4 TRIAL DESIGN

4.1 CANDIDATE SPECIFIC DESIGN

This study is a parallel group, randomised phase I trial and a parallel group, randomised, double-blind, placebo-controlled phase II trial to assess the safety, tolerability and effect of IV Favipiravir.

4.2 TRIAL PHASES

4.2.1 Dose finding (phase I)

Phase I will be open-label assessing escalating doses of IV Favipiravir compared with Standard of Care (SoC). Patients in phase I will be recruited in cohorts of 6 (randomised to IV Favipiravir: SoC in 2:1 allocation ratio), with review of safety and tolerability between cohorts.

Dose-finding will be carried out as per the Master Protocol (Master Protocol section 4.2.1).

4.2.1.1 Safety Review Committee (SRC) and dose escalation process

Refer to section 14.4 of the AGILE Master Protocol for definitions.

The SRC will review each dose cohort of 6 patients. Multiple doses of IV Favipiravir will be administered by intravenous (IV) infusion over 1 hour. Dosing regimen will be every 12 hours for 7 days duration. The starting dose will be 600 mg (BID), with a dose de-escalation possibility to a 300mg (BID) starting dose; and further dose escalations to 1200mg (BID), 1800mg (BID) and 2400mg (BID) are anticipated, with escalation and de-escalation guided by emerging safety data and decision by the SRC. Duration of monitoring will be 29 days post-first dose.

Membership of the SRC will be defined as per Study SRC charter to include the Chief Investigator (Saye Khoo) Chair of SRC, Candidate Chief Investigator, a principal investigator (PI) or delegate from the investigational site, one independent clinical member, , trial statistician and CRO trial manager or CRO

representative. At the chair's discretion, members who are unable to attend may be asked to provide comment to the chair to enable information to be shared.

The SRC will review all available safety data once the last participant recruited in the current cohort has Day 8 measures taken (where data is available beyond Day 8 for some participants, this will also be reviewed). This is inclusive of AE data, vital signs data, ECG data and clinical laboratory evaluations.

4.2.2 Efficacy Evaluation (Phase II)

Note this section differs from the AGILE Master Protocol.

Once the recommended phase II dose has been identified, phase II will start. In the first stage (Phase IIa) 66 patients are randomised between IV Favipiravir and placebo in a 1:1 allocation ratio. If the probability of the treatment effect (in terms of the viral load on Day 3) for the experimental arm being below 0 (i.e. in favour of IV Favipiravir) is more than 0.94, the study will stop for efficacy; if this probability is less than 0.3, then the study will stop for futility. Within this range, the trial will be expanded to stage 2 (Phase IIb) where an additional 132 patients are equally randomised between IV Favipiravir and placebo. Safety will be reviewed by a DMEC at regular intervals.

4.2.2.1 Data monitoring ethics committee (DMEC)

Refer to section 14.3 of the AGILE Master Protocol for definitions.

A DMEC will review results during the phase II stage evaluating safety and efficacy. Following each review, decision will be made to either:
- Stop evaluation due to high risk of harm
- Stop evaluation due to a low probability of IV Favipiravir having a meaningful effect
- Continue study
- Recommend IV Favipiravir for further evaluation in a definitive trial

There will be an interim analysis for harm and futility in phase II after 66 participants have been enrolled.

Stopping criteria for harm or futility
As per the master protocol, recruitment will cease if the probability that the risk of toxicity is at least 30% more than the control arm is 25% or more during phase I. Harm is defined by unacceptable toxicity as given by the Common Terminology Criteria for Adverse Events (CTCAE) criteria (see master protocol). Futility will be determined during phase II if after the initial 66 patients there is a less than 0.3 probability that the treatment effect is below 0.

Stopping criteria for efficacy
Efficacy will be established during phase II if after the initial 66 patients there is a greater than 0.94 probability that the change from baseline to Day 3 in viral load between arms is less than 0. The same criteria will also be used at the final analysis after 198 patients if the trial continues.

4.3 JUSTIFICATION FOR DOSE

The starting dose of IV Favipiravir administered in the protocol is lower than doses delivered in the oral formulation in extensive previous clinical trials.

A twice daily dose of 600mg over 1 hour infusion is proposed as the starting dose of the first-in-human study. Once the safety and pharmacokinetics of the starting dose is understood, the dose and

frequency will be escalated to find an optimized regimen for the treatment of COVID-19 patients. The justification for the starting dose is provided in the following paragraphs:

Justification of the Starting Dose
The safety of a single oral dose of favipiravir was studied up to 2400mg (BID) in healthy Japanese adults as a part of the QT study (#JP115), and the safety of a repeated oral dose up to 1800/800 mg twice a day for 22 days was investigated in a repeated dose safety study with healthy Japanese adults (#JP120). The Cmax (mean ± SD) observed in these studies were 116.11 ± 20.39 and 106.41 ± 22.67 µg/mL (Day 12 of administration), and the highest Cmax observed in these studies were 155 and 140 µg/mL, respectively. Two adverse events were observed in these studies; one event of nasopharyngitis in the single dose study subject, and one event of upper abdominal pain and increased blood uric acid in the repeated dose study. None of these were serious.

The oral and intravenous administration routes were compared in cynomolgus monkeys in a repeated dose safety study at 150 mg/kg twice daily (#SBL063-073). The Cmax (mean ± SD) after the first dose were 71.5 ± 29.4 µg/mL (oral) and 286±27 µg/mL (intravenous), indicating the Cmax after intravenous administration could be 4 times higher than that of oral route.

A Phase I, randomized, double-blind, placebo-controlled, single ascending-dose study to evaluate the pharmacokinetics, safety and tolerability of Injectable Favipiravir in healthy subjects in Japan was completed in 2021. Eight subjects in 5 cohorts (300, 600, 1200, 1800, 2400 mg) received active drug and two received placebo intravenously. At 300 to 2400 mg doses of favipiravir, the geometric mean Cmax ranged from 13.20 to 125.94 µg/mL (SD 8.8 µg/mL) , with a mean t1/2 of 1.5 hours at 300 mg, and 7.3 hours at the dose of 2400 mg.

No SAEs occurred in subjects who received favipiravir at doses of 300 to 2400 mg. Moderate adverse events occurred in 12.5% (1/8 subjects, 1 event of conjunctivitis) for 2400 mg favipiravir; however, this event was considered to be unrelated to favipiravir. All the other adverse events (n=8) were mild in severity. Of these, blood uric acid increased (1 event) and pyuria (1 event) were considered to be related to favipiravir. The Cmax after a single dose of 600 mg (60-minute infusion) in humans was a geometric mean of 8µg/mL, and including an empirical safety margin of 40% is well under the concentrations observed in higher doses given in #JP115 and #JP120 studies. In combination with the safety data generated from the IV formulation SAD study in healthy volunteers (300mg - 2400mg dose cohorts), a 600mg BID via intravenous infusion was proposed as the starting dose.

Dose escalation will be guided by safety data from the previous cohorts, with the magnitude of dose escalation being no more than a doubling from the previously tested dose. Doses were selected on the basis that our pharmacometric assessment (see figure above) indicated that doses ≥1200mg BID maintenance are expected to be required to maintain intracellular RTP metabolite concentration above its Km for the SARS-CoV-2 polymerase (Pertinez H., 2021).

4.4 DEFINITION OF END OF TRIAL

Refer to AGILE Master Protocol section 4.4

5 SELECTION AND ENROLMENT OF PATIENTS

This section is in addition to the AGILE Master Protocol

5.1 INCLUSION CRITERIA

The main trial (Phase I and II) inclusion criteria are outlined in the AGILE Master Protocol and listed below.

1. Adult in-patients (≥18 years) with laboratory confirmed COVID-19 infection by positive polymerase chain reaction (PCR) test within 7 days of randomisation
2. Ability to provide informed consent signed by study patient or legally acceptable representative
3. Women of childbearing potential (WOCBP) and male patients who are sexually active with WOCBP must agree to use a highly effective method of contraception (as outlined in section 5.4 below) from the first administration of trial treatment, throughout trial treatment and for the duration outlined in the candidate-specific trial protocol as well as addition 14 days for women and 7 days for men after the last dose of trial treatment.

Additional criteria specific to this candidate are:

4. Group A and B (moderate to severe disease). Patients with clinical status of Grades 4 (hospitalised, no oxygen therapy), 5 (hospitalised, oxygen by mask or nasal prongs), 6 (hospitalised, on non-invasive ventilation, or high flow oxygen) as defined by the WHO Clinical Progression Scale (WHO, 2020).
5. Less than or equal to 14 days from onset of COVID-19 symptoms

5.2 EXCLUSION CRITERIA

The main trial exclusion criteria are outlined in the master protocol as:

1. Alanine aminotransferase (ALT) and/or aspartate aminotransferase (AST) >5 times the upper limit of normal (ULN)
2. Stage 4 severe chronic kidney disease or requiring dialysis (i.e., estimated glomerular filtration (eGFR) rate <30 mL/min/1.73 m2)
3. Pregnant or breast feeding
4. Anticipated transfer to another hospital which is not a study site within 72 hours
5. Known Allergy to any study medication
6. Patients taking the following prohibited drugs within 30 days or 5 times the half-life (whichever is longer) of enrolment:

There are no known prohibited drugs with Favipiravir.

The following drugs should be used with caution when given with Favipiravir (Liverpool, n.d.):

- Pyrazinamide: Pyrazinamide administration with favipiravir examined possible renal urate transporter interactions. Pyrazinamide increased blood uric acid levels 2 to 9 mg/dL over baseline. The addition of favipiravir increased blood uric acid levels 4 to 11 mg/dL over baseline, indicating a moderate additive effect.

- Repaglinide: Favipiravir administration with repaglinide, an anti-diabetic agent that is extensively metabolized by CYP2C8 and CYP3A4, increased repaglinide plasma AUC 30 to 50% due to inhibition of CYP2C8.

- Theophylline: Theophylline administration with favipiravir increases plasma Cmax and AUC of favipiravir through xanthine oxidase (XO) interaction. The primary metabolite of theophylline is known to be metabolized by XO which is partially involved in metabolism of favipiravir.

- Famciclovir, Sulindac: Famciclovir and Sulindac are converted to active metabolite by Aldehyde Oxidase (AO). Favipiravir inhibits AO and decrease the concentration of active metabolite of Famciclovir and Sulindac.

- Paracetamol: Coadministration of paracetamol (650 mg once daily) and favipiravir (1200 mg twice daily or 800 mg twice daily) increased paracetamol Cmax and AUC by 3% and 16% (1200 mg doses) and by 8% and 14% (800 mg doses). The daily dose of paracetamol in adults should be no more than 3000 mg/day (rather than 4000 mg/day).

7. Patients participating in another clinical trial of an investigational medicinal product (CTIMP) and actively taking CTIMP within 30 days

5.3 SCREEN FAILURES

Refer to AGILE Master Protocol section 5.4

5.4 CONTRACEPTION

Refer to AGILE Master protocol section 5.5, with the following amendments:

- Women of childbearing potential (WOCBP) and male patients who are sexually active with WOCBP must agree to use a highly effective method of contraception from the first administration of trial treatment, throughout trial treatment and an additional 14 days for women and 7 days for men after the last dose of trial treatment
- Abstinence would be considered acceptable during the inpatient stay and post discharge; and while transitioning to a highly effective contraceptive measure after leaving the unit. A transition plan to include contraception measures required until the new method becomes effective should be agreed prior to hospital discharge.

5.5 REGISTRATION / RANDOMISATION PROCEDURES

Patients are registered on the trial database following consent. Randomisation is performed within the trial database when patients have completed screening and eligibility has been confirmed.

6 TREATMENTS

IV Favipiravir and placebo are considered investigational medicinal products for the purpose of this protocol. Favipiravir will be provided free of charge by Fujifilm Toyama Chemical Co., Ltd. for patients recruited to the trial. Saline placebo (for phase II) will be provided by the clinical site.

6.1 TREATMENT SCHEDULE

Participants will receive IV infusions of Favipiravir or standard of care (Phase I) or saline placebo (Phase II) twice daily up to 7 days whilst hospitalised.
N.B. placebo only in phase II

Arm Name	IV Favipiravir	Placebo
Type	Small molecule antiviral	Placebo
Dose Formulation	Lyophilised	Sterile 0.9% sodium chloride solution

Unit Dose Strength	600mg/vial	Not applicable
Dosage Level(s)	(300 only if dose de-escalated) 600, 1200, 1800, 2400 mg BID (maximum)	Same volume as active IMP
Route of Administration	Intravenous Infusion	Intravenous Infusion
Dosing instructions	Deliver over 1 hour Twice daily (every 12 hours (as per AGILE CST6 Acceptable Time Windows Working Instruction and protocol section 6.4)) for 7 days as inpatient.	Deliver over 1 hour Twice daily (every 12 hours (as per AGILE CST6 Acceptable Time Windows Working Instruction and protocol section 6.4)) for 7 days as inpatient
Packaging and Labelling	IMP will be provided in a single-use vial in an individual carton and labelled as required per country requirements.	Not applicable.
Current/Former Name(s) or Aliases(es)	T-705	Not applicable

6.2 IMP SUPPLY

Favipiravir will be provided to participating sites for clinical trial use. Refer to the Pharmacy Manual and the Favipiravir Site Dosing Instructions for details on supply, storage and preparation. Placebo will be sourced locally.

6.3 PRODUCT HANDLING, STORAGE AND STABILITY

The storage condition of the IMP should be stored in upright position and protected from light at room temperature.

The investigator or designee must confirm appropriate temperature conditions have been maintained during transit for all study intervention received and any discrepancies are reported and resolved before use of the study intervention.

Under normal conditions of handling and administration, study intervention is not expected to pose significant safety risks to site staff. Take adequate precautions to avoid direct eye or skin contact and the generation of aerosols or mists.

For futher information please refer to the pharmacy manual.

6.4 PREPARATION AND ADMINISTRATION

Please refer to the Pharmacy manual for more detailed information.

If patients miss a dose; record as missed dose if delayed by longer than four hours, and administer when next due. If able to administer within four hours, take as soon as able within that time and

record the actual time of dosing. If longer than four hours the dose is omitted, record missing dose administration.

6.5 ACCOUNTABILITY

Drug accountability logs will be provided to sites who will be responsible for maintaining records during the study as follows:

- Amount of study medication received
- Amount distributed to each patient
- Amount of unused drug returned or destroyed at the Sponsors request

In the event of necessary disposal of opened but wasted medication, the disposal should be documented appropriately (i.e. witnessed), in accordance with applicable local regulations, and GCP procedures. Copies of all completed drug accountability logs will be returned to CRO for compliance checks.

6.6 STUDY INTERVENTION COMPLIANCE

Participants will receive IMP or placebo (Phase II) directly from the investigator or delegated member of the clinical study team, under medical supervision. The date and start and stop times of the dose administered will be recorded in the source documents.

6.7 DOSE MODIFICATION

See Section 6.4 for instructions if dose is delayed beyond stipulated Treatment Schedule (section 6.1).

See Section 7 for instructions to discontinue study treatment for safety reasons.

6.8 CONTINUED ACCESS TO STUDY INTERVENTION AFTER THE END OF THE STUDY

COVID-19 is an acute illness and participants are not expected to need continued access to Favipiravir after the end of the study.

6.9 TREATMENT OF OVERDOSE

No specific treatment is recommended for an overdose. The treating physician will manage participants symptoms as clinically indicated.
In the event of an overdose, the treating physician should:

1. Contact the CRO and the Chief Investigator(s) immediately.
2. Closely monitor the participant for AE/SAE and laboratory abnormalities.
3. Document the quantity of the excess dose as well as the duration of the overdosing in the eCRF.

6.10 CONCOMITANT MEDICATIONS

Information on any medication received by the participant, including frequency and therapeutic indication from 30 days prior to randomisation and until Day 29 will be recorded in the electronic case report form (eCRF).
The Chief Investigator(s) should be contacted if there are any questions regarding concomitant or prior therapy.

6.11 PROHIBITED AND RESTRICTED THERAPIES DURING THE TRIAL

There are no known prohibited drugs with Favipiravir.
The following drugs should be used with caution when given with Favipiravir (Liverpool, n.d.):

- Pyrazinamide: Pyrazinamide administration with favipiravir examined possible renal urate transporter interactions. Pyrazinamide increased blood uric acid levels 2 to 9 mg/dL over

baseline. The addition of favipiravir increased blood uric acid levels 4 to 11 mg/dL over baseline, indicating a moderate additive effect.

- Repaglinide: Favipiravir administration with repaglinide, an anti-diabetic agent that is extensively metabolized by CYP2C8 and CYP3A4, increased repaglinide plasma AUC 30 to 50% due to inhibition of CYP2C8.

- Theophylline: Theophylline administration with favipiravir increases plasma Cmax and AUC of favipiravir through xanthine oxidase (XO) interaction. The primary metabolite of theophylline is known to be metabolized by XO which is partially involved in metabolism of favipiravir.

- Famciclovir, Sulindac: Famciclovir and Sulindac are converted to active metabolite by Aldehyde Oxidase (AO). Favipiravir inhibits AO and decrease the concentration of active metabolite of Famciclovir and Sulindac.

- Paracetamol: Coadministration of paracetamol (650 mg once daily) and favipiravir (1200 mg twice daily or 800 mg twice daily) increased paracetamol Cmax and AUC by 3% and 16% (1200 mg doses) and by 8% and 14% (800 mg doses). The daily dose of paracetamol in adults should be no more than 3000 mg/day (rather than 4000 mg/day).

6.12 PERMITTED CONCOMITANT MEDICATIONS

All medication that the participant is receiving as local, established standard of care for acute COVID-19 is permitted, as are stable concomittant medications that are being adminstered for past medical history. Any concerns regarding the acceptability of potential treatments should be discussed with the Chief Investigator(s).

7 DISCONTINUATION OF STUDY TREATMENT AND PATIENT DISCONTINUATION/WITHDRAWAL

Refer the Master AGILE protocol section 7.0

8 STUDY ASSESSMENT AND PROCEDURES

8.1 SCREENING PROCEDURES

Screening procedures to be carried out up to 1 day prior to randomisation and include:

- Informed Consent
- Eligibility Assessment
- Assessment using the WHO Clinical Progression Scale (WHO, 2020):
  0. Uninfected, no viral RNA detected
  1. Ambulatory mild disease, asymptomatic; viral RNA detected
  2. Ambulatory mild disease, symptomatic; independent
  3. Ambulatory mild disease, symptomatic; assistance needed
  4. Hospitalised moderate disease, no oxygen therapy (If hospitalised for isolation only, record stats as for ambulatory patient)
  5. Hospitalised moderate disease, oxygen by mask or nasal prongs
  6. Hospitalised severe disease, oxygen by NIV or high flow
  7. Hospitalised severe disease, intubation and mechanical ventilation $pO_2/FiO_2 \geq 150$ or $SpO_2/FiO_2 \geq 200$

8. Hospitalised severe disease, mechanical ventilation pO_2/FiO_2 <150 (SpO_2/FiO_2 <200) or vasopressors
9. Hospitalised severe disease, mechanical ventilation pO_2/FiO_2 <150 and vasopressors, dialysis, or ECMO
10. Dead

- Non-Ventilated Patients: National Early Warning Score 2 (NEWS2) Assessment:
    - Respiration rate
    - Oxygen saturation
    - Systolic blood pressure
    - Pulse rate
    - Level of consciousness or new confusion*
    - Temperature

      *The patient has new-onset confusion, disorientation and/or agitation, where previously their mental state was normal – this may be subtle. The patient may respond to questions coherently, but there is some confusion, disorientation and/or agitation. This would score 3 or 4 on the GCS (rather than the normal 5 for verbal response), and scores 3 on the NEWS system.
- 12 lead ECG and 10 second ECG rhythm strip (≥5 min supine), to be performed during screening, or on baseline day 1 prior to first dose.
- Full blood count
- Urea and electrolytes
- Estimated GFR
- Liver Function Tests
- Uric acid
- Urinary analysis
- Women of childbearing potential: Pregnancy test (serum)
- Medical history (including COVID-19 history e.g. symptom onset)
- Concomitant medication and standard of care review
- Height/supine length and weight (*(to be performed during screening, or on baseline day 1 prior to first dose. Can be taken from current admission.*
- Assessment of oxygen use (low flow, high flow)
- Assessment of mechanical ventilation use (Only if supplemental oxygen not being administered, lowest oxygen saturation over the last 24 hours to be recorded as part of baseline/screening)
- Demographics review
- Adverse Event Assessment (from consent)
- Targeted Physical examination

The following assessment, listed in the Master Protocol, is not required at screening in this candidate specific trial protocol:

- Ventilated Patients: Quick Sepsis-Related Organ Dysfunction Assessment (qSOFA) Score

8.2 TRIAL PROCEDURES

Participants will receive treatment up to 7 days when hospitalised for COVID-19 and cease when they have been discharged from hospital. Visits outlined in the schedule of events will take place as in-patients when in hospital or out-patient. If discharged participants are not able to return after discharge for clinic visits , follow-up should be done by phone call to the patient and a

minimised criteria will apply to perform AE assessment, concomititant medication and patient status.

Note that this CST visit schedule differs from the Master Protocol and is the schedule to be used for all patients enrolled onto this candidate specific trial.

8.2.1 Baseline Visit (Day 1, day of randomisation)

The following assessments are to be carried out on the day of randomisation. Patients should commence treatment on the day of randomisation (i.e. Day 1).

- Assessment using the WHO Clinical Progression Scale (WHO, 2020):
    0. Uninfected, no viral RNA detected
    1. Ambulatory mild disease, asymptomatic; viral RNA detected
    2. Ambulatory mild disease, symptomatic; independent
    3. Ambulatory mild disease, symptomatic; assistance needed
    4. Hospitalised moderate disease, no oxygen therapy (If hospitalised for isolation only, record stats as for ambulatory patient)
    5. Hospitalised moderate disease, oxygen by mask or nasal prongs
    6. Hospitalised severe disease, oxygen by NIV or high flow
    7. Hospitalised severe disease, intubation and mechanical ventilation pO_2/FiO_2 ≥150 or SpO_2/FiO_2 ≥200
    8. Hospitalised severe disease, mechanical ventilation pO_2/FiO_2 <150 (SpO_2/FiO_2 <200) or vasopressors
    9. Hospitalised severe disease, mechanical ventilation pO_2/FiO_2 <150 and vasopressors, dialysis, or ECMO
    10. Dead
- Non-Ventilated Patients: National Early Warning Score 2 (NEWS2) Assessment:
    - Respiration rate
    - Oxygen saturation
    - Systolic blood pressure
    - Pulse rate
    - Level of consciousness or new confusion*
    - Temperature

      *The patient has new-onset confusion, disorientation and/or agitation, where previously their mental state was normal – this may be subtle. The patient may respond to questions coherently, but there is some confusion, disorientation and/or agitation. This would score 3 or 4 on the GCS (rather than the normal 5 for verbal response), and scores 3 on the NEWS system.

  Vital Signs are collected prior to dose administration, + 30 minutes, end of infusion +1 hour post infusion for Dose 1 only.

- Concomitant medication and standard of care review
- Assessment of oxygen use (low flow, high flow)
- Assessment of mechanical ventilation use ((Only if supplemental oxygen not being administered, lowest oxygen saturation over the last 24 hours to be recorded as part of baseline/screening))
- Adverse event assessment
- SARS-CoV-2 nose/throat swab for storage for future translational research
- SARS-CoV-2 surveillance NP swab

- Chest X-Ray (if clinically indicated)

In addition, for candidate specific trial protocol the following are required:

- Review inclusion and exclusion criteria and confirm eligibility
- Randomisation
- The following is required if not performed within 24 hours of Baseline *(results are required prior to dosing only if clinically indicated)*:
  - Full blood count
  - Urea and electrolytes
  - Estimated GFR
  - Liver Function Tests
  - Uric acid
  - Urinary analysis
- Pre-Dose Blood collection for translational research
- Blood samples for PK (Blood and dried blood spot) - Day 1 timepoints of pre-dose, 0-1 hr, 2-4 hr and 6-12 hr post completion of first infusion
- Non-plasma (tears, saliva, nasal secretions) – Day 1 timepoints of 6-12 hr post completion of the first infusion
- PBMC sample - Day 1 timepoints of 6-12 hr post completion of first infusion
- Safety bloods (if clinically indicated)
- Drug administration of Favipiravir (or placebo for Phase II) IV BID dose for 7 days, by infusion over up to 1 h, to be administered in hospital
- Targeted physical examination

8.2.2 Treatment

Phase I

Participants will be randomised to receive IV Favipiravir or SoC only (2:1) on Day 1. Refer to section 6 of the CST6 protocol for further information.

Phase II

Participants will be randomised to receive IV Favipiravir or saline placebo (alongside SoC) on Day Refer to section 6 of the CST6 protocol for further information.

8.2.3 Daily Day 2 through to Day 14:

Daily Day 2 through to Day 14: when in hospital or via telephone contact if leaves hospital before day 8 to document: adverse events and concomitant medication. First 5 days of treatment, daily SARS-CoV-2 surveillance NP swab**.** The following as per SoC and if clinically indicated if in hospital:

- Full blood count, U&Es, Uric acid, estimated GFR, LFTs
- Vital signs
- Assessment of oxygen use
- Targeted physical exam

8.2.4 Specific Assessments on Days 3, 5, 8 and 11 (±1 day)

The following assessments are to be carried out on 3, 5, 8 and 11 (NB day of randomisation and start date of treatment is Day 1).

- SARS-CoV-2 surveillance NP swab
- Full blood count
- Urea and electrolytes

- Estimated GFR
- Liver Function Tests
- Uric acid
- Concomitant medication and standard of care review
- AE assessment
- Translational swab (Days 3 and 5 only)

In addition, for CST6 candidate specific trial protocol the following are required:

- Assessment using the WHO Clinical Progression Scale (WHO, 2020):
    0. Uninfected, no viral RNA detected
    1. Ambulatory mild disease, asymptomatic; viral RNA detected
    2. Ambulatory mild disease, symptomatic; independent
    3. Ambulatory mild disease, symptomatic; assistance needed
    4. Hospitalised moderate disease, no oxygen therapy (If hospitalised for isolation only, record stats as for ambulatory patient)
    5. Hospitalised moderate disease, oxygen by mask or nasal prongs
    6. Hospitalised severe disease, oxygen by NIV or high flow
    7. Hospitalised severe disease, intubation and mechanical ventilation $pO_2/FiO_2 \geq 150$ or $SpO_2/FiO_2 \geq 200$
    8. Hospitalised severe disease, mechanical ventilation $pO_2/FiO_2 < 150$ ($SpO_2/FiO_2 < 200$) or vasopressors
    9. Hospitalised severe disease, mechanical ventilation $pO_2/FiO_2 < 150$ and vasopressors, dialysis, or ECMO
    10. Dead
- Non-Ventilated Patients: National Early Warning Score 2 (NEWS2) Assessment:
    - Respiration rate
    - Oxygen saturation
    - Systolic blood pressure
    - Pulse rate
    - Level of consciousness or new confusion*
    - Temperature

    *The patient has new-onset confusion, disorientation and/or agitation, where previously their mental state was normal – this may be subtle. The patient may respond to questions coherently, but there is some confusion, disorientation and/or agitation. This would score 3 or 4 on the GCS (rather than the normal 5 for verbal response), and scores 3 on the NEWS system.
- Assessment of oxygen use (low flow, high flow, mechanical flow, mechanical ventilation use) (only if supplemental oxygen is not administered) – daily whilst in hospital
- Targeted Physical examination
- Blood (plasma and DBS) taken for PK assessments on Day 3 (pre-dose, 0-1 hr, 2-4 hr, 6-12 hr post completion of first infusion) and Day 5 (0-1hr, 6-12 hr post completion of first infusion)
- Tears, saliva, nasal swab PK assessments - Day 3 timepoints at 6-12 hr post completion of first infusion
- PBMC sample - Day 3 & Day 5 timepoints at 6-12 hr post completion of first infusion
- Blood taken for translational research

8.2.5 *Day 15 (±2 Days)*

The following assessments are to be carried out on Day 15 (±2days) (NB day of randomisation and start date of treatment is Day 1). This differs from the Master protocol as +1 day is permitted to this visit date.

- SARS-CoV-2 surveillance NP swab
- Assessment using the WHO Clinical Progression Scale (WHO, 2020):
    0. Uninfected, no viral RNA detected
    1. Ambulatory mild disease, asymptomatic; viral RNA detected
    2. Ambulatory mild disease, symptomatic; independent
    3. Ambulatory mild disease, symptomatic; assistance needed
    4. Hospitalised moderate disease, no oxygen therapy (If hospitalised for isolation only, record stats as for ambulatory patient)
    5. Hospitalised moderate disease, oxygen by mask or nasal prongs
    6. Hospitalised severe disease, oxygen by NIV or high flow
    7. Hospitalised severe disease, intubation and mechanical ventilation pO_2/FiO_2 ≥150 or SpO_2/FiO_2 ≥200
    8. Hospitalised severe disease, mechanical ventilation pO_2/FiO_2 <150 (SpO_2/FiO_2 <200) or vasopressors
    9. Hospitalised severe disease, mechanical ventilation pO_2/FiO_2 <150 and vasopressors, dialysis, or ECMO
    10. Dead
- Non-Ventilated Patients: National Early Warning Score 2 (NEWS2) Assessment:
    - Respiration rate
    - Oxygen saturation
    - Systolic blood pressure
    - Pulse rate
    - Level of consciousness or new confusion*
    - Temperature

      *The patient has new-onset confusion, disorientation and/or agitation, where previously their mental state was normal – this may be subtle. The patient may respond to questions coherently, but there is some confusion, disorientation and/or agitation. This would score 3 or 4 on the GCS (rather than the normal 5 for verbal response), and scores 3 on the NEWS system.
- Full blood count
- Urea and electrolytes
- Estimated GFR
- Liver Function Tests
- Uric acid
- Concomitant medication and standard of care review
- AE assessment

- Assessment of oxygen use (low flow, high flow, mechanical flow, mechanical ventilation use) (only if supplemental oxygen is not administered) – daily whilst in hospital
    - Chest X-Ray (if clinically indicated)

In addition, for candidate specific trial protocol the following are required:

- Targeted physical exam
- Blood collection for translational research

Day 29 (±2 days)

The following assessments are to be carried out on Day 29 (+2 days) (NB day of randomisation and start date of treatment is Day 1).

- SARS-CoV-2 surveillance NP swab
- Assessment using the WHO Clinical Progression Scale (WHO, 2020):
  0. Uninfected, no viral RNA detected
  1. Ambulatory mild disease, asymptomatic; viral RNA detected
  2. Ambulatory mild disease, symptomatic; independent
  3. Ambulatory mild disease, symptomatic; assistance needed
  4. Hospitalised moderate disease, no oxygen therapy (If hospitalised for isolation only, record stats as for ambulatory patient)
  5. Hospitalised moderate disease, oxygen by mask or nasal prongs
  6. Hospitalised severe disease, oxygen by NIV or high flow
  7. Hospitalised severe disease, intubation and mechanical ventilation pO_2/FiO_2 ≥150 or SpO_2/FiO_2 ≥200
  8. Hospitalised severe disease, mechanical ventilation pO_2/FiO_2 <150 (SpO_2/FiO_2 <200) or vasopressors
  9. Hospitalised severe disease, mechanical ventilation pO_2/FiO_2 <150 and vasopressors, dialysis, or ECMO
  10. Dead
- Non-Ventilated Patients: National Early Warning Score 2 (NEWS2) Assessment:
  - Respiration rate
  - Oxygen saturation
  - Systolic blood pressure
  - Pulse rate
  - Level of consciousness or new confusion*
  - Temperature

    *The patient has new-onset confusion, disorientation and/or agitation, where previously their mental state was normal – this may be subtle. The patient may respond to questions coherently, but there is some confusion, disorientation and/or agitation. This would score 3 or 4 on the GCS (rather than the normal 5 for verbal response), and scores 3 on the NEWS system.
- Full blood count
- Urea and electrolytes
- Estimated GFR
- Liver Function Tests
- Uric acid
- Concomitant medication and standard of care review
- AE assessment
- Targeted physical exam
- Urine pregnancy test for WOCBP
- Urinalysis

8.3 SAMPLE REQUIREMENTS

This section differs from the Master Protocol and is specific to this candidate.

Samples will be collected in accordance with the laboratory manual and schedule of observations and procedures (Section 8.2). Sponsor may store samples for up to 25 years after the end of the study to achieve study objectives.

Pharmacodynamics

8.3.1 Viral Swabs

Patients will be asked to give consent for use of their PCR nasal swabs collected at every clinic visit including baseline and all treatment and follow-up visits. This is mandatory for phase I and II.

The same samples used for SARS-CoV-2 PCR will be used, so collection and labs will be as standard clinical practice.

Patients will have the option to consent for an additional PCR nasal swab to be collected on days 1, 3 and 5 for use in future translational research.

8.3.2 Pharmacokinetics

Phase I

All patients will be asked to give consent for collection of blood, tears, saliva, and nasal secretions for paired measurement of drug concentrations. Samples will be collected after first dose, on Day 1, Day 3 and Day 5 of treatment according to the timing windows below, recognising that a pragmatic, opportunistic sampling approach is required to sample patients receiving non-invasive ventilatory support (NIV) during periods when NIV is paused as part of clinical care.

Matrix	Sample Type/Collection Tube	Day 1 & Day 3				Day 5	
		pre-dose	Time post-completion of infusion (hr)				
			0-1 hr	2-4 hr	6-12 hr	0-1 hr	6-12 hr
**Plasma**[1]	K2 EDTA tube (2 mL)	X	X	X	X	X	X
**Dried Blood Spot**[1]	Hemasep card (2 x spots)	X[a]	X[a]	X[a]	X[a]	X[a]	X[a]
**Tears**[1]	2 x Schirmer Tear Test Strips				X		
**Saliva**[1]	Salivette tube				X		
**Nasal secretions**[1]	2 x nasal swabs (SAM)				X		
**PBMC**[1]	1 x CPT (8 mL)				X		X

[1] For patients who are on NIV, sampling is pragmatic and opportunistic, and may be omitted according to physician discretion
[a] 2 x 100 µL whole blood taken from K2 EDTA tube, prior to centrifugation, and spot onto DBS card

PK Sample Kit
PK 1
PK 2
PK3

PK Sample Kit 1 (PK1): for collection and processing of whole blood [plasma + DBS] on Day 1 & Day 3 [pre-dose, 0-1 hr, 2-4 hr] and Day 5 [0.1 hr]

PK Sample Kit 2 (PK2): for collection and processing of PBMC and all plasma and non-plasma samples on Day 1 & Day 3 [6-12 hr timepoint]

PK Sample Kit 3 (PK3) = for collection and processing of plasma + DBS and PBMC on Day 5 [6-12 hr timepoint]

Phase II

For phase II, patients may choose to give optional consent for the PK sub study. Sparse sampling (plasma only; maximum of 4 time points per patient) will be undertaken over the duration of treatment, with opportunistic sampling timed to coincide with any pause in delivery of NIV that is scheduled as part of clinical care.

All samples will need to be processed and frozen for batch shipment to Bioanalytical Facility, University of Liverpool.

Further details are provided in the lab manual.

Safety Laboratory Evaluations

This information is in addition to the Master Protocol.

Blood samples will be collected for safety laboratory evaluations at the timepoints indicated above. Additional clinical laboratory evaluations will be performed at other times if judged to be clinically appropriate, required by hospital policy (e.g. MRSA and SARS-CoV-2 screening) or if the on-going review of the data suggests a more detailed assessment of safety laboratory evaluations is required. An investigator will perform a clinical assessment of all clinical laboratory data. Samples will need to be processed at site and will be analysed by the local site laboratory.

8.4 DEVIATIONS AND SERIOUS BREACHES

Refer to the AGILE Master Protocol section 8.4, with addition of CRO to be informed of all deviations and serious breaches. Candidate specific information relating to serious breaches reporting is listed in section 8.4 which supersedes information in the corresponding section of the AGILE Master Protocol.

Additionally, the site and Sponsor will adopt all reasonable measures to record data in accordance with the protocol. It is accepted, however, that some minor variations may occur in the day to day conduct of the study. All such deviations will be documented in the Investigator Site File and Trial Master File (recorded by CRO), with the reasons for their occurrence. Where appropriate, the deviations will be detailed in the clinical study report.

All serious breaches of Good Clinical Practice and/or the trial protocol will be reported to the regulatory authorities and other organisations, as required in the Medicines for Human Use (Clinical Trials) Regulations 2004, as amended but no later than 7 days of the Sponsor/CRO becoming aware

CST-6 reporting responsibilities as per CST-6 Trial Matrix Allocation (TAM).

9 SAFETY

9.1 SAFETY EVENT REPORTING:

Refer to the AGILE Master protocol for definitions. As per the Master the below information has been added specific to the candidates under investigation in this protocol.

9.1.1 Dose Limiting Toxicity (DLT)

Dose limiting toxicities will be defined as CTCAE v5 Grade ≥3 adverse events, independently assessed to be possibly or probably related to the IMP as per SRC charter. The assessment of relatedness is included in the DLT definition for this CST due to the potential for a high frequency of CTCAE v5 Grade ≥3 adverse events related to disease or co-morbidity in these cohort of hospitalised patients and the desire to avoid labelling potential treatments as unsafe due to misclassifying non-treatment related toxicities.

9.2 REPORTING WINDOWS

All AEs will be collected up to Day 29 post-dose. SAEs will be collected from dose administration through to Day 29 follow-up visit. However, any SAEs assessed as related to study participation (e.g., study intervention, protocol-mandated procedure, invasive tests or change in existing therapy) will be recorded from the time participant consents to participate in the study.

Each time there is a change in grade of an adverse event this should be recorded on a separate log line on the adverse event form on the eCRF. Refer to the eCRF Guidance on how to report this on Rave. The investigator should notify the trial sponsor of any death or adverse event occurring at any time after a patient has discontinued or terminated trial participation that may reasonably be related to this trial.

9.3 ADVERSE EVENT TERM AND SEVERITY GRADE

Refer to the AGILE Master Protocol section 9.3

9.4 SERIOUSNESS

Refer to the AGILE Master Protocol section 9.4

Additionally, all SAEs must be reported immediately by the PI or delegate at the participating centre to the CRO using study specific SAE form.

9.4.1 Exceptions

Note that this section differs from the AGILE Master Protocol

For the purposes of this trial, the following SAEs do not require reporting to SCTU using the Serious Adverse Event Report Form:

- Death due to disease progression of COVID-19 – This is the condition for which the participant is being treated. Unless death is considered related to the candidate.
- Any other as appropriate e.g. SAEs occurring prior to trial treatment/intervention, that are not considered to be related to trial procedures
- SAEs occurring prior to the first dose of the candidate, that are not considered to be related to trial procedures

9.5 CAUSALITY

Refer to the AGILE Master Protocol section 9.5

9.6 EXPECTEDNESS ASSESSMENT:

There are no expected events associated with Favipiravir, hence all SARs are to be considered SUSARs.

Refer to the Investigator's Brochure for the current Reference Safety Information including ADRs and to the AGILE Master protocol for additional safety considerations.

Name of Product	IB	Section/Table No.	Manufacturer	Date of text revision DD-MMM-YYYY
T-705 (Favipiravir)	3	Section 6	Fujifilm Toyama Chemical Co., Ltd	02-FEB-2022

9.7 REPORTING PROCEDURES

Refer to the AGILE Master Protocol section 9.7 for full details of reporting procedures. Candidate specific information relating to pregnancy reporting is listed in section 9.7.1 which supersedes information in the corresponding section of the AGILE Master Protocol.

9.7.1 Pregnancy

- Details of all pregnancies in female participants will be collected after the start of study intervention and until Week 24 via telephone consultation.
- If a pregnancy is reported, the investigator will record pregnancy information on the appropriate form and submit it to sponsor or designee within 24 hours of learning of the female participant pregnancy. While pregnancy itself is not considered to be an AE or SAE, any pregnancy complication or elective termination of a pregnancy for medical reasons will be reported as an AE or SAE.
- Abnormal pregnancy outcomes (e.g., spontaneous abortion, fetal death, stillbirth, congenital anomalies, ectopic pregnancy) are considered SAEs and will be reported as such.
- The participant will be followed to determine the outcome of the pregnancy. The investigator will collect follow-up information on the participant and the neonate and the information will be forwarded to the sponsor or designee.
- Any post-study pregnancy-related SAE considered reasonably related to the study intervention by the investigator will be reported to the sponsor or designee. While the investigator is not obligated to actively seek this information in former study participants, he or she may learn of an SAE through spontaneous reporting.
- Any female participant who becomes pregnant while participating in the study will be withdrawn from the study.

9.8 CLINICAL RESEARCH ORGANISATION (CRO) RESPONSIBILITIES FOR SAFETY REPORTING TO REC

PHARMExcel (CRO) will have expedited reporting responsibilities to REC as the CRO for this trial.
Urgent Safety Measure (USM) reporting will be undertaken by SCTU.
DSUR preparation will be undertaken as per the CST-6 TAM and reporting undertaken by SCTU.

9.9 CLINICAL RESEARCH ORGANISATION (CRO) RESPONSIBILITIES FOR SAFETY REPORTING TO MHRA

PHARMExcel (CRO) will have expedited reporting responsibilities to MHRA as the CRO for this trial.
Urgent Safety Measure (USM) reporting will be undertaken by SCTU.
DSUR preparation will be undertaken as per the CST 6 TAM and reporting undertaken by SCTU.

9.10 EMERGENCY UNBLINDING (PHASE II ONLY)

This section is in addition to the Master Protocol.

Patient allocation will be provided to unblinded pharmacy staff via an IWRS. The randomisation code will be retained by the system.
Deaths and serious adverse events (SAE) will be reviewed in a blinded manner. If a cause of death or SAE is unexpected (i.e. not listed in the RSI approved section of the IB) and considered drug related the trial statistician and Quality and Regulatory team (SCTU) will be able to unblind the patient's treatment assignment.
24hr emergency unblinding service will be provided through the IWRS system. Sites must have tested procedure for out-of-hours emergency unblinding.
In the case of adverse events in which patient care would vary dependent on treatment allocation, emergency unblinding may be performed by unblinded pharmacy staff or in cases where it is the site's local policy for on-call doctors to unblind, this will be permitted. In these situations, discussion with the Chief Investigator is not required. If patient allocation is disclosed, sites should report to SCTU the date, time, reason for unblinding, name of person requesting the code break, and name of person breaking the code using the trial specific Emergency Unblinding Notification Form. Unblinding reports should be filed in the patient's medical records at site and in the trial master file (without disclosing the treatment allocation).
The Trial Manager or Senior Trial Manager will ensure that the Statistician and Senior Statistician are fully informed of all cases of unblinding. A participant may continue in the study if that participant's intervention assignment is unblinded, providing there are no safety issues. Potential continuation should be the decision of the treating physician.

All further details are outlined in the study specific procedure for unblinding.

10 STATISTICS AND DATA ANALYSES

10.1 METHOD OF RANDOMISATION

Note this differs from the AGILE Master Protocol and is the method of randomisation to be used for this specific candidate.

For phase I, participants will be randomised 2:1 to IV Favipiravir or SoC.

For phase II, participants will be randomised in a 1:1 ratio to IV Favipiravir or placebo.

10.2 SAMPLE SIZE

Phase I sample size

The sample size for phase I is not fixed. A total of up to 5 cohorts of 6 participants are planned to be dosed. However, two additional cohorts may be added. The dose in these cohorts will not exceed 2400mg (BID).

Phase II sample size

A sample size of up to 99 patients on active treatment and up to 99 on placebo, with an interim analysis after the first 66 patients is estimated to provide the 80% probability detecting the true treatment effect of 0.5 $\log_{10}$ reduction in viral load, and over 90% probability for the effect of 0.6 $\log_{10}$ reduction, (assuming a SD for change from baseline in log10 viral load of 1.5 in each group) while controlling the probability of claiming superiority while there is no difference (type I error) below 10%.

10.3 STATISTICAL ANALYSIS PLAN (SAP)

Refer to AGILE Master Protocol section 10.3

10.3.1 Phase I

Study population

An evaluable patient for the study will be all patients randomised to Favipiravir, who have had at least one dose or randomised to standard of care.

Dose Cohorts

The first phase 1 cohort (6 patients) will be randomised to starting dose of 600mg BID dose or standard of care (2:1 ratio), with subsequent planned dose escalation to 1200mg BID, to a maximum dose of 2400mg BID and a possible de-escalation dose of 300mg BID if necessary. Escalation will occur only in the case of acceptable safety and tolerability at the next lowest dose (modelled as per the Master Protocol and additional requirements for this CST). De-escalation will be guided by DLTs and SRC decisions.

For each dose level, the first two eligible participants enrolled will be randomized 1:1 to Favipiravir or standard of care. These sentinel participants will be dosed and monitored for 24 hours post-dose. If there has been no serious adverse event considered by the Investigator to be at least possibly related to the study drug and no other significant safety concern, the remainder of the cohort will be dosed. This will be documented and reviewed by the local site PI(s) with oversight from the CI.

Decision Making

Dose escalation will be based on review of safety data up to Day 8 on the safety population. Doses are deemed to be safe if the risk of dose-limiting toxicity (DLT) being at least 30% larger in IV Favipiravir than standard of care is less than 25%. Dose de-escalation for the starting dose will be reviewed by the SRC and guided by their decision depending on DLTs.

A two parameter Bayesian dose escalation model will be used to recommend the next cohort dose or the recommended phase II dose. The model will target a safe dose with an additional DLT risk of 20% (the target interval of 15-25%) above standard of care.

Analyses

Please refer to the AGILE master protocol phase I analysis section 10.3.1. Bayesian credible intervals will be the 95% equal-tail intervals.

10.3.2 Phase II

Note this section differs from the AGILE Master Protocol.

Once the recommended phase II dose, as defined by the safety and tolerability data in Phase I has been identified, Phase II will start. In the first stage 66 patients will be equally randomised between Favipiravir or placebo. If the probability of the treatment effect (in terms of the viral load on Day 3) for the experimental arm being below 0 (i.e., in favour of Favipiravir) is more than 0.94, the study will stop for efficacy; if this probability is less than 0.3, then the study will stop for futility. Within this range, the trial will be expanded to stage 2 where an additional 132 patients are equally randomised between Favipiravir and placebo. Safety will be reviewed by a DMEC at regular intervals.

Efficacy population

An evaluable patient for the study will be all patients randomised to Favipiravir or placebo (intention-to-treat).

Analysis
Patients will be analysed according to the treatment to which they were randomised (efficacy population). In addition, a supplementary analysis of the primary endpoint will be conducted in all patients for whom there were no major protocol deviations that could impact treatment efficacy (to be defined in the statistical analysis plan).

The primary efficacy endpoint in phase II of the SARS-CoV-2 viral load (in log10) will be analysed using a Bayesian repeated measures model including all post-randomisation assessments up to and including Day 3. The model will adjust for treatment, baseline viral load, visit timepoint and interaction of treatment by visit, with a subject-specific random-effect and unstructured variance-covariance matrix. A non-informative (vague) prior will be used for all model parameters. Viral load data will be $\log_{10}$-transformed prior to analysis with treatment differences expressed on the $\log_{10}$ scale. If 94% of the posterior distribution of the treatment effect is below 0 (i.e. in favour of Favipiravir), the study will conclude the treatment is efficacious, at the interim or the final analysis.

For any deaths in the trial, the change in viral load will be assumed to be zero from the last measured timepoint. To assess the impact of missingness in the primary analysis due to missing data that are not missing at random (including death) sensitivity analyses will be performed. Details will be provided in the statistical analysis plan.

Once the last participant recruited completes the Day 29 assessment the data will be unblinded to allow a primary readout of this endpoint. Participants will continue to complete the remaining scheduled assessments and will not be unblinded at an individual level.

Further details on analysis methods for other endpoints will be detailed in a detailed SAP, which will be finalised and signed off prior to database lock.

11 REGULATORY

11.1 CLINICAL TRIAL AUTHORISATION

Refer to AGILE Master Protocol section 11

12 ETHICAL CONSIDERATIONS

Refer to AGILE Master Protocol section 12

13 SPONSOR

The trial sponsor is University of Liverpool.
SCTU, Chief Investigator, a Clinical Research Organisation and other appropriate organisations have been delegated specific duties by the Sponsor and this is documented in the trial task allocation matrix (TAM).

The duties assigned to the trial sites (NHS Trusts or others taking part in this trial) are detailed in the Non-Commercial Agreement.

13.1 INDEMNITY

Refer to AGILE Master Protocol section 13.1

13.2 FUNDING

Fujifilm Toyama Chemical Co., Ltd, the manufacturer for the investigational medicinal product (IMP), is providing the study drug - Favipiravir. This will be further supported by other funding streams such as the Medical Research Council, Wellcome Trust and The Therapeutic Taskforce.

None of the doctors or other staff conducting the research are being paid directly for recruiting patients into the study.

14 TRIAL OVERSIGHT GROUPS

Refer to AGILE Master Protocol section 14 and relevant CST-6 specific Charters.

15 DATA MANAGEMENT

Refer to AGILE Master Protocol section 15.

15.1 AUDITS AND INSPECTIONS

Note this differs from the AGILE Master Protocol

The trial may be participant to inspection and audit by the sponsor (under their remit as Sponsor), CRO (as the Sponsor's delegate) and other regulatory bodies to ensure adherence to the principles of GCP, Research Governance Framework for Health and Social Care, applicable contracts/agreements and national regulations.

Southampton Clinical Trials Unit will notify of any MHRA inspection at Sponsor, site, CRO or CTU.

16 DATA SHARING REQUESTS FOR RESULTS THAT ARE AVAILABLE IN THE PUBLIC DOMAIN

Refer to AGILE Master Protocol section 16.

17 MONITORING

Refer to the AGILE Master Protocol section 17 and CST-6 specific Monitoring Plan.

18 RECORD RETENTION AND ARCHIVING

Refer to the AGILE Master Protocol section 18.

19 PUBLICATION POLICY

Refer to AGILE Master Protocol section 19.

20 REFERENCES

Bialek, e. a., 2020. *Severe outcoomes among patients with coronavirus disease 2019 (COVID-19),* s.l.: Morbidity and Mortality Weekly Report.

Chen, e. a., 2020. pidemiological and clinical characteristics of 99 cases of 2019 novel coronavirus pneumonia in Wuhan, China: a descriptive study.. *The Lancet.*

Chen, X. e. a., 2020. Serological evidence of human infection with SARS-CoV-2: a systematic review and meta-analysis. *MedRxIV.*

Choi, S. e. a., 2020. Antiviral activity and safety of remdemsivir against SARS-CoV-2 infection in human pluripotent stem cell-derived cardiomyocytes.. *Antiviral Res,* Volume 184, p. 104955.

Choy, K. e. a., 2020. Remdesivir, lopinavir, emetine, and homoharringtonine inhibit SARS-CoV-2 replication in vitro. *Antiviral Res,* Volume 178, p. 104786.

Driouich, J. e. a., 2021. Favipiravir antiviral efficacy against SARS-CoV-2 in a hamster model.. *Europe PMC,* 12(1), p. 1735.

Ferguson, e. a., 2020. Coronavirus fatality rate estimated by Imperial scientists. *The Lancet - Infectious Diseases,* 20(6), pp. 669-677.

FUFIFILM Toyama Chemical Co., L., 2020. *Clinical Study report JP324 Phase 3 Study,* Japan: FUJIFILM Toyama Chemical Co., Ltd..

Furuta, Y. K. T. N. T., 2017. Favipiravir (T705), a broad spectrum inhibitor of viral RNA polymerase.. *Japan Academy,* 93(7), pp. 449-463.

Hassanipour, S. A.-Z. M. A. B. e. a., 2021. The efficacy and safety of Favipiravir in treatment of COVID-19: a systematic review and meta-analysis of clinical trials. *Science Rep,* 11(11022), pp. 90551-6.

Hattori, S. e. a., 2020. GRL-0920, an Indole Chloropyridinyl Ester, Completely Blocks SARS-CoV-2 Infection. *mBio,* 11(4).

Hopkins, J., 2022. *Coronavirus Resource Center.* [Online]
Available at: https://coronavirus.jhu.edu/map.html
[Accessed September 2022].

Ivashchenko, A. e. a., 2020. *AVIFAVIR for Treatment of Patients with Moderate COVID-19: Interim Results of a Phase II/III Multicenter Randomized Clinical Trial,* s.l.: Clin Infec Dis.

Kaptein, S. J. F. e. a., 2020. Favipiravir at high doses has potent antiviral activity in SARS-CoV-2–infected hamsters, whereas hydroxychloroquine lacks activity. *PNAS,* 117(43), pp. 26955-26965.

Klok, e. a., 2020. A matter of time: duration and choice of venous thromboprophylaxis in patients diagnosed with COVID-19. *British Journal of Hospital Medicine,* 81(5).

Liverpool, U. o., n.d. *Covid-19 Drug Interactions.* [Online]
Available at: https://www.covid19-druginteractions.org/
[Accessed June 2021].

Nguyen TH, G. J. A. X. L. C. e. a., 2017. JIKI Study group. Favipiravir pharmacokinetics in Ebola-infected patients of the JIKI trial reveals concentrations lower than targeted. *PLoS Negl Trop Dis,* 11(2).

Pertinez H., R. R. K. S. O. A., 2021. Pharmacokinetic modelling to estimate intracellular favipiravir ribofuranosyl-5'-triphosphate exposure to support posology for SARS-CoV-2.. *MedRxiv .*

Sissoko, D. e. a., 2016. Experimental Treatment with Favipiravir for Ebola Virus Disease (the JIKI Trial): A Historically Controlled, Single-Arm Proof-of-Concept Trial in Guinea. *PLOS Medicine.*

Suzanne, J. K. F. e. a., 2020. Favipiravir at high doses has potent antiviral activity in SARS-CoV-2-infected hamsters, whereas hyroxychloroquine lacks activity.. *Proceedings of the national academy of sciences,* 117(43), pp. 26955-26965.

Udwadia, Z. e. a., 2020. Efficacy and safety of favipiravir, an oral RNA-dependent RNA polymerase inhibitor, in mind-to-moderate COVID-19: A randomized, comparative, open-label, multicenter, Phase 3 clinical trial.. *International Journal of Infectious Diseases,* 103(2021), pp. 62-71.

Wang, M. C. R. Z. L. e. a., 2020. Remdesevir and chloroquine effectively inhibit the recently emerged novel coronavirus (2019-nCoV) in vitro. *Cell Res,* Volume 30, pp. 269-271.

Wang, M. e. a., 2020. Remdesivir and chloroquine effectively inhibit the recently emerged novel coronavirus (2019-nCoV) in vitro.. *Cell Res,* 30(3), pp. 269-271.

WHO, 2020. WHO Working Group on the Clinical Characterisation and Management of COVID-19 infection (2020). A minimal common outcome measure set for COVID-19 clinical research. *Lancet Infect Dis,* pp. 192-197.

Wiersinga, e. a., 2020. Pathophysiology, Transmission, Diagnosis, and Treatment of Coronavirus Disease 2019 (COVID-19). *Journal of American Medical Association,* 324(8), pp. 782-793.

Zhou, e. a., 2020. Clinical course and risk factors for mortality of adult inpatients with COVID-19 in Wuhan, China: a retrospective cohort study. *The Lancet,* 395(10229), pp. 1054-1062.

21 SUMMARY OF SIGNIFICANT CHANGES TO THE CANDIDATE SPECIFIC TRIAL PROTOCOL

Protocol date and version	Summary of significant changes
V1.0 03-MAR-2022	First Version
V2.0 26-May-22	Clarification of exploratory objective, update of PK sample table and section 8.3, update of RSI to new IB edition
V3.0 16 Sept 2022	Amendment to population and inclusion criteria to include Grade 4 (hospitalised, no oxygen therapy), clarification Day 1 is day of randomisation (baseline), update to background and rationale, update to references
V4.0 13 Jan 2023	Amendment to clarify primary endpoint and objectives, reference to acceptable window working instruction within the schedule of observation and procedures, clarity on dose administration in section 6, update to SRC, update on deviation and serious breaches reporting processes, clarification on DLT definition and update to CRO responsibilities.

Agile 6_TMF_Protocol_V4.0 Clean_13Jan23

Final Audit Report 2023-03-24

Created:	2023-03-16
By:	Robyn Edwards (robyn.edwards@pharmexcel-cro.com)
Status:	Signed
Transaction ID:	CBJCHBCAABAAssBuEt9z0H6BxBCeAJcUNqPE3Mx-18k7

"Agile 6_TMF_Protocol_V4.0 Clean_13Jan23" History

Document created by Robyn Edwards (robyn.edwards@pharmexcel-cro.com)
2023-03-16 - 2:23:42 PM GMT

Document emailed to khoo@liverpool.ac.uk for signature
2023-03-16 - 2:25:30 PM GMT

Email viewed by khoo@liverpool.ac.uk
2023-03-16 - 2:25:45 PM GMT

Signer khoo@liverpool.ac.uk entered name at signing as Saye Khoo
2023-03-16 - 2:26:01 PM GMT

Document e-signed by Saye Khoo (khoo@liverpool.ac.uk)
Signature Date: 2023-03-16 - 2:26:03 PM GMT - Time Source: server

Document emailed to tom.fletcher@lstmed.ac.uk for signature
2023-03-16 - 2:26:04 PM GMT

Email viewed by tom.fletcher@lstmed.ac.uk
2023-03-16 - 2:31:25 PM GMT

Signer tom.fletcher@lstmed.ac.uk entered name at signing as T E Fletcher
2023-03-16 - 2:38:21 PM GMT

Document e-signed by T E Fletcher (tom.fletcher@lstmed.ac.uk)
Signature Date: 2023-03-16 - 2:38:23 PM GMT - Time Source: server

Document emailed to g.o.griffiths@soton.ac.uk for signature
2023-03-16 - 2:38:25 PM GMT

Email viewed by g.o.griffiths@soton.ac.uk
2023-03-23 - 8:14:55 PM GMT

New document URL requested by g.o.griffiths@soton.ac.uk

2023-03-23 - 8:14:59 PM GMT

Signer g.o.griffiths@soton.ac.uk entered name at signing as G Griffiths

2023-03-23 - 8:15:41 PM GMT

Document e-signed by G Griffiths (g.o.griffiths@soton.ac.uk)

Signature Date: 2023-03-23 - 8:15:43 PM GMT - Time Source: server

Document emailed to kwilding@liverpool.ac.uk for signature

2023-03-23 - 8:15:45 PM GMT

Email viewed by kwilding@liverpool.ac.uk

2023-03-24 - 7:46:24 AM GMT

Signer kwilding@liverpool.ac.uk entered name at signing as Karen Wilding

2023-03-24 - 7:46:48 AM GMT

Document e-signed by Karen Wilding (kwilding@liverpool.ac.uk)

Signature Date: 2023-03-24 - 7:46:50 AM GMT - Time Source: server

Agreement completed.

2023-03-24 - 7:46:50 AM GMT

Names and email addresses are entered into the Acrobat Sign service by Acrobat Sign users and are unverified unless otherwise noted.

Adobe Acrobat Sign
