# Supplementary material for: Optimal Dose and Safety of Intravenous Favipiravir in Hospitalized Patients With COVID‐19: A Dose‐Escalating, Randomized Controlled Phase Ib Study
Source: Clin Pharmacol Ther. 2026 Mar 18;119(6):1650–61. doi: 10.1002/cpt.70261 (PMC13156351; doi:10.1002/cpt.70261)
Supplement: Supplementary file 2 — Data S2. Summary of parameter estimates for favipiravir 1‐compartment IV infusion POP‐PK model fitting [file CPT-119-1650-s003.docx]

| Parameter (units) | Estimate (95% CI) | Inter-individual  variance (ω^2^, log  parameters) | Shrinkage (%) |
| --- | --- | --- | --- |
| Cl­_70kg_ (L/h) | 3.21 (2.08, 4.95) | 0.765 | -1.1 |
| V_d, 70kg_ (L) | 29.3 (25.3, 33.9) | 0.036 | 46.1 |
| Proportional error (σ^2^) | 0.202 |  |  |
| Half-life (h) | 9.32 (1.08, 36.12)^a^ |  |  |
| Cl­_70kg_: Clearance for an individual with a bodyweight of 70kg  V_d, 70kg_: Volume of distribution for an individual with a bodyweight of 70kg  ^a^Bootstrap calculation using 10^5^ samples of bodyweight, clearance and volume of distribution | | | |

S2. Summary of parameter estimates for favipiravir 1-compartment IV infusion POP-PK model fitting
