# Supplementary material for: Optimal Dose and Safety of Intravenous Favipiravir in Hospitalized Patients With COVID‐19: A Dose‐Escalating, Randomized Controlled Phase Ib Study
Source: Clin Pharmacol Ther. 2026 Mar 18;119(6):1650–61. doi: 10.1002/cpt.70261 (PMC13156351; doi:10.1002/cpt.70261)
Supplement: Supplementary file 3 — Data S3. PK parameters generated from popPK model [file CPT-119-1650-s001.docx]

S3. Summary of favipiravir plasma pharmacokinetic parameters predicted using the pop-PK model for a 79.8 kg bodyweight individual

| Day | Dose (mg, twice daily) | C_max_ (µg/mL) | AUC_0-last_ (h*µg/mL) | AUC_0-24_(h*µg/mL) |
| --- | --- | --- | --- | --- |
| Day 1 | 600 | 19.3959 | 133.8645 | 306.5328 |
|  | 1200 | 38.7919 | 267.729 | 613.0656 |
|  | 1800 | 58.1878 | 401.5935 | 919.5985 |
|  | 2400 | 77.5838 | 535.4579 | 1226.1313 |
| Day 3 | 600 | 26.4805 | 186.6399 | 373.4817 |
|  | 1200 | 52.961 | 373.2798 | 746.9634 |
|  | 1800 | 79.4414 | 559.9197 | 1120.4451 |
|  | 2400 | 105.9219 | 746.5596 | 1493.9267 |
| Day 5 | 600 | 26.5173 | 186.9145 | 373.83 |
|  | 1200 | 53.0347 | 373.8289 | 747.6599 |
|  | 1800 | 79.552 | 560.7434 | 1121.4899 |
|  | 2400 | 106.0693 | 747.6578 | 1495.3198 |
| C_max_: maximum measured concentration  AUC_0-last_: area under the concentration-time curve from pre-dose to last measured sample  AUC_0-24:_ area under the concentration-time curve over 24 hours following pre-dose sample | | | | |
