# Supplementary material for: Optimal Dose and Safety of Intravenous Favipiravir in Hospitalized Patients With COVID‐19: A Dose‐Escalating, Randomized Controlled Phase Ib Study
Source: Clin Pharmacol Ther. 2026 Mar 18;119(6):1650–61. doi: 10.1002/cpt.70261 (PMC13156351; doi:10.1002/cpt.70261)
Supplement: Supplementary file 4 — Data S4. Prior Parameters for the Randomised Bayesian Dose‐Escalation Model for CST‐6 [file CPT-119-1650-s005.docx]

Prior Parameters for

the Randomised Bayesian Dose-Escalation Model for CST-6

(Revised in Feb 2022 to change the starting dose for cohort 1)

**Prior Parameters and Simulation Results**

The dose-escalation model is

$$\varphi(\tilde{d_{j}},\theta_{1},\theta_{2})=\frac{exp(\theta_{1}+ \theta_{2}\tilde{d_{j}})}{1+ exp(\theta_{1}+ \theta_{2}\tilde{d_{j}})}$$

where $\tilde{d_{j}}$ are the standardized levels obtained through prior estimates of the DLE probabilities ${\hat{p_{j}}}^{(0)}$:

$$\tilde{d_{j}}=\frac{\mathrm{logit}\left( {\hat{p_{j}}}^{\left( 0 \right)} \right)-{\hat{\theta_{1}}}^{(0)}}{{\hat{\theta_{2}}}^{(0)}}$$

where ${\hat{\theta_{1}}}^{(0)}$, ${\hat{\theta_{2}}}^{(0)}$ are prior point estimates of the model parameters, with $\tilde{d_{j}}$= 0.

For the setting of CST-6 with 5 experimental doses, the prior parameters were calibrated in April 2021. Due to information from another trial, the decision was made by the chief investigator to change the starting dose from dose 1 to dose 2, keeping dose 1 as a de-escalation dose if required. Prior parameters were re-calibrated in February 2022 using the scenarios given in Table 1 over a grid of values of each of the hyperparameters. The scenarios for calibration and evaluation were chosen given the intention to proceed to Phase II trial with this compound only with the highest dose while safeguarding the patients.

The following parameters were found to result in good operating characteristics (see Table 1)

over all five scenarios while also resulting in intuitive escalation/de-escalation decisions (see page 2): ${(\theta}_{1},log(\theta_{2}))\sim N(\mu,\Sigma)$ where $(\mu_{1},\mu_{2})=(logit\left( 0.1 \right),-0.2$)^T^ is the vector of means and

$$\Sigma=\left[ \begin{matrix} 0.3 & 0 \\ 0 & 0.5 \end{matrix} \right]$$

using a spacing between prior probabilities of 0:15, the vector of standardized dose levels is $\tilde{d}$ =

(0.00, 1.05, 1.70, 2.28, 2.90, 3.74). The proportion of each dose selection under 5 scenarios with N = 36 (6 Cohorts) are given in Table 1.

**Table 1:** Proportion of selection for each dose under the calibrated model for N = 36. The correct selections are in bold. Results are based on 2,000 simulations.

|  |  | d_1_ | d_2_ | d_3_ | d_4_ | d_5_ | Stop for safety |
| --- | --- | --- | --- | --- | --- | --- | --- |
| Scenario 1 | Toxicity: | **0.3** | 0.45 | 0.60 | 0.70 | 0.80 |  |
|  | Selection: | **51.2%** | 13.1% | 1.7% | 0.0% | 0.0% | 34.1% |
| Scenario 2 | Toxicity: | 0.15 | **0.30** | 0.45 | 0.60 | 0.75 |  |
|  | Selection: | 25.5% | **49.5%** | 16.0% | 1.8% | 0.1% | 7.3% |
| Scenario 3 | Toxicity: | 0.12 | 0.15 | **0.30** | 0.45 | 0.60 |  |
|  | Selection: | 4.3% | 29.8% | **48.3%** | 14.8% | 1.1% | 1.8% |
| Scenario 4 | Toxicity: | 0.11 | 0.12 | 0.15 | **0.30** | 0.45 |  |
|  | Selection: | 1.0% | 5.6% | 33.2% | **46.2%** | 12.9% | 1.3% |
| Scenario 5 | Toxicity: | 0.11 | 0.12 | 0.13 | 0.15 | **0.30** |  |
|  | Selection: | 0.2% | 1.6% | 9.7% | 30.6% | **57.5%** | 0.6% |

**Escalation Strategies**

Illustration of the escalation decision after the first cohort of patients (2 patients on the control,

4 patients on the second dose of 600mg) if 0 DLTs on control, and X DLTs (out of 4 patients) on the active compound. For each case, a table with the following quantities is presented:

- Estimated Toxicity (Mean)
- Overdose Probability = P (p_j_ – p_0_ ≥ 30%)
- Target Probability = P (p_j_ – p_0_ ∈ (15%, 25%))

Note that the Probabilities are formulated in terms of the *excessive toxicity* (over the control).

**Prior Distribution (no trail participants):** start at dose d_2_:

|  | SoC | d_1_ | d_2_ | d_3_ | d_4_ | d_5_ |
| --- | --- | --- | --- | --- | --- | --- |
| Mean Toxicity | 11.07 | 27.51 | 39.59 | 48.98 | 57.46 | 66.73 |
| Overdose | 0.00 | 15.27 | 36.27 | 51.95 | 64.60 | 76.55 |
| Target | 0.00 | 16.71 | 19.15 | 17.04 | 13.81 | 9.93 |

0 DLTs: Next recommended dose is d_3_ (d_4_ is too toxic at 31.11%):

|  | SoC | d_1_ | d_2_ | d_3_ | d_4_ | d_5_ |
| --- | --- | --- | --- | --- | --- | --- |
| Mean Toxicity | 8.42 | 14.47 | 20.19 | 26.24 | 33.28 | 42.96 |
| Overdose | 0.00 | 0.11 | 4.84 | 16.28 | 31.11 | 49.61 |
| Target | 0.00 | 4.16 | 17.93 | 23.54 | 23.65 | 19.78 |

1 DLT: Next recommended dose is to stay on d_2_ (d_3_ is too toxic at 36.9%):

|  | SoC | d_1_ | d_2_ | d_3_ | d_4_ | d_5_ |
| --- | --- | --- | --- | --- | --- | --- |
| Mean Toxicity | 9.97 | 19.41 | 28.43 | 37.30 | 46.62 | 57.88 |
| Overdose | 0.00 | 1.15 | 16.96 | 36.88 | 54.79 | 71.33 |
| Target | 0.00 | 13.78 | 26.04 | 23.94 | 18.70 | 12.71 |

2 DLTs: Next recommended dose is d_1_:

|  | SoC | d_1_ | d_2_ | d_3_ | d_4_ | d_5_ |
| --- | --- | --- | --- | --- | --- | --- |
| Mean Toxicity | 11.34 | 26.52 | 40.53 | 52.53 | 63.30 | 74.22 |
| Overdose | 0.00 | 7.75 | 42.16 | 64.63 | 78.42 | 88.20 |
| Target | 0.00 | 28.06 | 23.99 | 15.51 | 9.81 | 5.46 |

3 DLTs: Terminate the trial (d_1_ is too toxic at 32.4%):

|  | SoC | d_1_ | d_2_ | d_3_ | d_4_ | d_5_ |
| --- | --- | --- | --- | --- | --- | --- |
| Mean Toxicity | 12.27 | 37.73 | 57.90 | 71.36 | 80.96 | 88.71 |
| Overdose | 0.00 | 32.44 | 74.51 | 87.98 | 93.81 | 97.12 |
| Target | 0.00 | 30.23 | 11.79 | 5.60 | 2.91 | 1.36 |
