# Supplementary material for: Optimal Dose and Safety of Intravenous Favipiravir in Hospitalized Patients With COVID‐19: A Dose‐Escalating, Randomized Controlled Phase Ib Study
Source: Clin Pharmacol Ther. 2026 Mar 18;119(6):1650–61. doi: 10.1002/cpt.70261 (PMC13156351; doi:10.1002/cpt.70261)
Supplement: Supplementary file 5 — Data S5. Reporting checklist for dose escalation or de‐escalation trial [file CPT-119-1650-s004.docx]

Reporting checklist for dose escalation or de- escalation trial.

Based on the CONSORT-DEFINE guidelines.

Complete this checklist by entering the page numbers from your manuscript where readers will find each of the items listed below.

Your article may not currently address all the items on the checklist. Please modify your text to include the missing information. If you are certain that an item does not apply, please write "n/a" and provide a short explanation.

Upload your completed checklist as an extra file when you submit to *Clinical Pharmacology & Therapeutics*.

In your methods section, say that you used the CONSORT-DEFINE reporting guidelines, and cite them as:

Yap C, Solovyeva O, de Bono J, Rekowski J, Patel D, Jaki T et al, Enhancing reporting quality and impact of early phase dose-finding clinical trials: CONSORT Dose-finding Extension (CONSORT- DEFINE) guidance. BMJ 2023;383:e076387.

|  |  | Reporting Item | Page  Number |
| --- | --- | --- | --- |
| **Title and Abstract** |  |  |  |
| Title | #1a | Identification as an early phase dose-finding (eg, first-in- human, dose escalation or de-escalation, phase 1, phase 1/2, expansion, dose titration) and, if applicable, randomised trial in the title or abstract | 1 |
| Abstract | #1b | Structured summary of trial design, methods, results, and conclusions (for specific guidance, see CONSORT- DEFINE for abstracts) | 3 |
| **Introduction** |  |  |  |
| Background and objectives | #2a.1 | Description of research question(s) and justification for undertaking the trial, including summary of relevant clinical studies (published and unpublished) examining benefits and harms for each intervention | 4,5 |
|  | #2a.2 | Summary of key findings from relevant non-clinical or preclinical research | 4,5 |

|  | #2a.3 | Summary of findings from previously generated preclinical and translational studies to support any planned biomarker substudies (where applicable) | n/a – no substudy |  |  |
| --- | --- | --- | --- | --- | --- |
|  | #2b | Specific objectives (eg, relating to safety, activity, pharmacokinetics, pharmacodynamics, recommended dose(s)) | 5 |  |  |
| **Methods** |  |  |  |  |  |
| Trial design | #3a.1 | Description of trial design elements, such as dose escalation or de-escalation strategy, number of treatment groups, allocation ratio if relevant, and details of any prespecified trial adaptations | 5/6 |  |  |
|  | #3a.2 | Trial design schema to show the flow of major transition points (eg, dose escalation to dose expansion, phase 1 to phase 2, single ascending dose to multiple ascending dose) | Fig.1 |  |  |
|  | #3a.3 | Statistical methods or rationale underpinning the trial design | 5/6/7 |  |  |
|  | #3a.4 | Starting dose(s) with rationale | 5 |  |  |
|  | #3a.5 | Range of planned dose levels with rationale | 5 |  |  |
|  | #3a.6 | Presentation of planned dose levels (eg, as a diagram, table, or infographic), where applicable | 6 (in text) |  |  |
|  | #3a.7 | Skipping of dose level(s), if applicable | n/a – no skips |  |  |
|  | #3a.8 | Planned cohort size(s) (eg, fixed, flexible, adaptive) | 5 |  |  |
|  | #3a.9 | Dose allocation method within a dose level (including sequence and interval between dosing of participants, eg, sentinel or staggered dosing) | 6 |  |  |
|  | #3a.10 | Dose expansion cohort(s), if applicable, with rationale | n/a – no DE |  |  |
|  | #3a.11 | Criteria for progression to the next part of the trial (eg, phase 1 to phase 2, single ascending dose to multiple ascending dose), where applicable | n/a – phase I only |  |  |
|  | #3b | Important changes to the design or methods after trial commencement (eg, insertion of unplanned additional doses) outside the scope of the prespecified adaptive design features, with reasons | 5,6 |  |  |
| Participants | #4a | Eligibility criteria for participants | 5,6 |  |  |
|  | #4b | Settings and locations where the data were collected | 5 |  |  |

| Interventions | #5a | Interventions for each dose level (within each group) with sufficient details to allow replication, including administration route and schedule showing how and when they were actually administered | 6 |  |  |
| --- | --- | --- | --- | --- | --- |
|  | #5b | Criteria for dose discontinuation, dose modifications, and dosing delays of allocated interventions for a given trial participant (eg, dose change in response to harms, participant request, or improving or worsening disease) | 7 |  |  |
| Outcomes | [#6a](https://www.goodreports.org/reporting-checklists/consort/info/#6a) | Primary and secondary outcomes, including the specific measurement variable, analysis metric, method of aggregation, and time point for each outcome.  Explanation of the clinical relevance of chosen outcomes is strongly recommended. Any other outcomes used to inform prespecified adaptations should be described with the rationale | 7 |  |  |
|  | #6b | Any unplanned changes to trial outcomes after the trial commenced, with reasons | 6 |  |  |
| Sample size | #7a | Estimated number of participants (minimum, maximum, or expected range) needed to address trial objectives and how it was determined, including clinical and statistical assumptions supporting any sample size and operating characteristics | 5 |  |  |
|  | #7b | Prespecified interim decision making criteria or rules that guided the trial adaptation process (eg, dosing decision to escalate or de-escalate); prespecified and actual timing and frequency of interim data reviews and the information to inform trial adaptations | 7 |  |  |
| **Randomization (if applicable)** | | |  |  |  |
| Sequence generation | #8a | Method used to generate the random allocation sequence. | 6 |  |  |
|  | #8b | Type of randomisation; details of any restrictions (such as blocking and block size); any prespecified adaptive assignment rules or algorithm leading to adjustments in the allocation ratio, including timing and frequency of updates; any changes to the allocation rule following trial adaptation decisions | 6 |  |  |
| Allocation concealment mechanism | #9 | Mechanism used to implement the random allocation sequence (such as sequentially numbered containers), describing any steps taken to conceal the sequence until interventions were assigned | 6 |  |  |

| Implementation | #10 | Who generated the allocation sequence, who enrolled participants, and who assigned participants to | 6 |  |  |
| --- | --- | --- | --- | --- | --- |
| Blinding | #11a | interventions  If done, who was blinded after assignment to interventions (for example, participants, care providers, those assessing outcomes) and how. | n/a – open label |  |  |
|  | #11b | If relevant, description of the similarity of interventions | n/a – vs SoC |  |  |
| Statistical methods | #12a.1 | Statistical methods for primary and secondary outcomes and any other outcomes used to make prespecified adaptations | 8 |  |  |
|  | #12a.2 | For the implemented adaptive design features, statistical methods used for estimation (eg, safety, dose(s), treatment effects) and to make inferences | 8 |  |  |
|  | #12b | Statistical methods for additional analyses (eg, subgroup and adjusted analyses, pharmacokinetics or pharmacodynamics, biomarker correlative analyses) | 10 |  |  |
|  | #12c | Analysis population(s) (eg, evaluable population for dose- finding, safety population) | 8 |  |  |
|  | #12d | Strategies for handling intercurrent events occurring after treatment initiation (eg, how dosing adjustments were handled) that can affect either the interpretation or the existence of the measurements associated with the clinical question of interest, and any methods to handle missing data | 8, S1 |  |  |
| **Results** |  |  |  |  |  |
| Participant flow diagram (strongly recommended) | #13a | For each group, the number of participants who were assigned to each dose level at each interim analysis (eg, for dosing decisions), received intended treatment, and were analysed for the primary outcome and, if applicable, any other outcomes used to inform prespecified adaptations | 9, Fig 1 |  |  |
|  | #13b | For each group, losses and exclusions after allocation to each dose level, together with reasons | 9, fig 1 |  |  |
| Recruitment | #14a | Dates defining the periods of recruitment and follow-up | 9 |  |  |
|  | #14b | Why the trial ended or was stopped | 9 |  |  |
|  | #14c | Trial adaptation decisions made (including on what basis they were made, and when) in light of the prespecified decision making criteria and observed, accrued data | 9 |  |  |

| Baseline data | #15 | Baseline demographic and clinical characteristics across each dose level within each group, where appropriate | Tab 1 (18) |  |  |
| --- | --- | --- | --- | --- | --- |
| Numbers analysed | #16 | For each group, the number of participants (denominator) included in each analysis across each dose level, and whether the analysis was by original assigned interventions | Fig 1 |  |  |
| Outcomes and estimation | #17a | For each primary and secondary outcome, results for each dose level within each group, and the estimated effect size and its precision, if applicable | 9,10, fig 2 |  |  |
|  | #17b | For binary outcomes, presentation of both absolute and relative effect sizes is recommended | Fig 2 |  |  |
|  | #17c | Report interim results used to inform interim decision making such as dose escalation, de-escalation, or staying at the same dose | Fig 2, 9 |  |  |
| Ancillary analyses | #18 | Results of any other analyses performed, including subgroup analyses and adjusted analyses, distinguishing pre-specified from exploratory | 10,11 |  |  |
| Harms | #19 | All important harms (eg, adverse events or effects, toxicities) reported by dose level in each group (for specific guidance, see CONSORT for harms) | 9, Tab 2 (19) |  |  |
| **Discussion** |  |  |  |  |  |
| Limitations | #20 | Trial limitations, addressing sources of potential bias, imprecision, and, if relevant, multiplicity of analyses | 11,12 |  |  |
| Generalisability | #21 | Generalisability (external validity, applicability) of the trial findings | 12 |  |  |
| **Other information** |  |  |  |  |  |
| Interpretation | #22 | Interpretation consistent with results, balancing benefits and harms, and considering other relevant evidence | 12 |  |  |
| Registration | #23 | Registration number and name of trial registry | 4 |  |  |
| Protocol | #24 | Where the full trial protocol can be accessed, if available | S1 |  |  |
| Funding | #25 | Sources of funding and other support (such as supply of drugs), role of funders | 2 |  |  |
| Data Monitoring | #26a | Composition of any decision making or safety review committee or group; summary of its role and reporting structure; statement of whether it is independent from the sponsor and competing interests; and reference to where further details can be found (such as in a charter or protocol) | S1 |  |  |

|  | #26b | Description of who had access to interim results and made the interim and final decision to terminate the trial (or part(s) of the trial, eg, end of dose escalation), and measures to safeguard the confidentiality of interim information | 7 |  |  |
| --- | --- | --- | --- | --- | --- |
| Dissemination | #27 | Specify, if applicable, whether and when results (such as safety and/or activity) were reported externally (eg, through scientific presentations, journal publication, or the  trial website) while the trial (or part(s) of the trial) was still ongoing | 2 |  |  |
